# How are health technology assessment bodies responding to the assessment challenges posed by cell and gene therapy?

**DOI:** 10.1186/s12913-023-09494-5

**Published:** 2023-05-13

**Authors:** Michael Drummond, Oriana Ciani, Giulia Fornaro, Claudio Jommi, Eva Susanne Dietrich, Jaime Espin, Jean Mossman, Gerard de Pouvourville

**Affiliations:** 1 Centre for Health Economics, University of York, York, UK; 2 CERGAS, SDA Bocconi School of Management, Milan, Italy; 3 University of Bonn Pharmaceutical Institute, Bonn, Germany; 4 Andalusian School of Public Health, Andalusia, Spain; 5 Patient Representative and Visiting Senior Research Associate in the Medical Technology Research Group, LSE Health, London School of Economics, London, UK; 6 ESSEC, Paris, France

**Keywords:** Cost-effectiveness analysis, Advanced therapy medicinal products, Health technology assessment, Managed entry agreements, Reimbursement

## Abstract

**Background:**

The aims of this research were to provide a better understanding of the specific evidence needs for assessment of clinical and cost-effectiveness of cell and gene therapies, and to explore the extent that the relevant categories of evidence are considered in health technology assessment (HTA) processes.

**Methods:**

A targeted literature review was conducted to identify the specific categories of evidence relevant to the assessment of these therapies. Forty-six HTA reports for 9 products in 10 cell and gene therapy indications across 8 jurisdictions were analysed to determine the extent to which various items of evidence were considered.

**Results:**

The items to which the HTA bodies reacted positively were: treatment was for a rare disease or serious condition, lack of alternative therapies, evidence indicating substantial health gains, and when alternative payment models could be agreed. The items to which they reacted negatively were: use of unvalidated surrogate endpoints, single arm trials without an adequately matched alternative therapy, inadequate reporting of adverse consequences and risks, short length of follow-up in clinical trials, extrapolating to long-term outcomes, and uncertainty around the economic estimates.

**Conclusions:**

The consideration by HTA bodies of evidence relating to the particular features of cell and gene therapies is variable. Several suggestions are made for addressing the assessment challenges posed by these therapies. Jurisdictions conducting HTAs of these therapies can consider whether these suggestions could be incorporated within their existing approach through strengthening deliberative decision-making or performing additional analyses.

**Supplementary Information:**

The online version contains supplementary material available at 10.1186/s12913-023-09494-5.

## Introduction

Cell and gene therapies comprise the majority of the products that the European Medicines Agency designates Advanced Therapy Medicinal Products (ATMPs) [1]. While they offer patients potentially transformational gains in health, these therapies also pose issues for healthcare payers in all jurisdictions. Much has already been written about the distinctive characteristics of these new therapies, and the need (or otherwise) for new health technology assessment (HTA) methods, [2–9]. There has also been discussion of how these characteristics might shape price and reimbursement negotiations [10, 11] and managed entry agreements [12, 13]. Finally, the literature has also explored the cost-effectiveness profile of cell and gene therapies [14–16] and the variation in the reimbursement or coverage decisions made in different jurisdictions [17–19].

Many of the assessment challenges relating to cell and gene therapies apply to rare disease treatments more generally, and most of the current cell and gene therapies are for rare conditions. However, in the case of cell and gene therapies the challenges are compounded by the potentially transformational nature of the health gain and the potential long-term nature of the ‘cure’, which is subject to considerable uncertainty.

The issue of how HTA bodies are responding to the assessment challenges posed by cell and gene therapies has received relatively lower attention. HTA methods are partly conditioned by the evidence currently available and new methods often imply the need for new types of evidence. For example, the clinical evidence for cell and gene therapies may be sparse in relation to that available for most pharmaceuticals, the economic benefits produced by cell and gene therapies may require additional evidence to demonstrate them and many of the innovative payment models being proposed may require further evidence generation post product launch.

In addition, although some of the papers in the current literature argue for a different approach to the HTA of cell and gene therapies, most HTA bodies prefer to use a standardized approach for all health technologies. In this context, the paper by Drummond et al. [4] argues that HTA bodies could apply their standardized approach, but should give specific attention to some of the particular challenges posed by cell and gene therapies.

Therefore, the aims of this research were: i). to provide a better understanding of the specific evidence needs for assessment of the clinical and cost-effectiveness of cell and gene therapies, ii) to explore the extent that the relevant categories of clinical evidence, economic evidence and evidence post-launch are considered in the HTA processes in several jurisdictions, iii) to identify the issues in the generation and use of evidence that require further discussion and consideration.

## Methods

To achieve the aims above, we undertook i) a targeted review of the literature on the clinical and economic evidence needs for these therapies, and ii) an in-depth analysis of HTA reports from 8 major jurisdictions for 9 cell and gene therapies in 10 indications, together with any associated publicly available documents on managed entry agreements and post-launch evidence requirements.

### Targeted literature review

Our starting point for the targeted literature review was the paper by Drummond et al. [4]. We selected this paper, which discussed the arguments for a new reference case for the HTA of gene therapies, because it contained the most extensive discussion to date of the implied clinical and economic evidence needs. However, the paper is essentially expert opinion based on a limited review of the literature available at the time. Therefore, rather than accept the suggestions made in that paper without question, we felt a further literature review was necessary to determine whether any new issues had arisen, or whether any relevant issues had been overlooked.

Given that there is already a substantial literature on the assessment challenges posed by cell and gene therapies, including several targeted or systematic reviews [15, 20–22], we first reviewed the references in the most recently published systematic review [22] to identify any papers focussing on evidence requirements. We selected 18 papers for detailed review from these sources. Then we followed a single-round ‘pearl growing’, or ‘snowballing’ approach, via PubMed, starting from the paper by Drummond et al. 2019 until the end of February 2022 [4]. We excluded abstracts and conference proceedings and included peer-reviewed papers if they discussed existing or new challenges related to assessment, together with recommendations about the evidence needed to perform this assessment. In total there were 36 citations of the Drummond et al. paper during this period. Of these, 25 were retrieved as peer-reviewed papers, and 20 were used in the review. Five were excluded as they did not deal with the challenges of assessing cell and gene therapies. Additional references to post-launch requirements were also retrieved from the grey literature. The papers identified were critically appraised by two co-authors independently, and referenced and summarised narratively according to the framework proposed in the Drummond et al. checklist.

### Analysis of HTA reports

For the selection and inclusion of HTA reports in our analysis, we identified HTA evaluation reports for cell and gene therapies from 7 HTA bodies, and one regulatory agency that also performs HTAs, published prior to end of July 2021. The agencies were based in the 5 largest European countries, plus the US and Canada: Agencia Española de Medicamentos y Productos Sanitarios (AEMPS) in Spain, Agenzia Italiana del Farmaco (AIFA) in Italy, Canadian Agency for Drugs and Technologies in Health (CADTH) in Canada, Gemeinsamer Bundesausschuss (G-BA) in Germany, Haute Autorité de Santé (HAS) in France, Institute for Clinical and Economic Review (ICER) in USA, National Institute for Health and Care Excellence (NICE) in England, and Scottish Medicines Consortium (SMC) in Scotland. The agencies selected were also representative of different approaches to conducting HTAs, some using the cost per QALY approach and others relying on an interpretation of the clinical data alone (see Table 1).

**Table 1:** HTA reports analyzed

	NICE (GB)	ICER (US)	CADTH (CA)	SMC (GB)	AIFA (IT)	HAS (FR)	G-BA (DE)	AEMPS (ES)
Kymriah DLBCL	X			X	X	X	X	X
Kymriah ALL	X	X			X	X	X	X
Yescarta	X	X		X	X	X	X	X
Luxturna	X	X	X	X		X	X	X
Strimvelis	X							
Imlygic	X						X	X
Alofisel	X					X	X	X
Provenge	X						X	
Glybera						X	X	
Zolgensma	X	X	X	X	X	X	X	

Key: *AEMPS* Agencia Española de Medicamentos y Productos Sanitarios, *AIFA* Agenzia Italiana del Farmaco, *ALL* acute lymphoblastic leukemia, *CADTH* Canadian Agency for Drugs and Technologies in Health, *DLBCL* diffuse large B-cell lymphoma, *G-BA* Gemeinsamer Bundesausschuss, *HAS* Haute Autorité de Santé, *ICER* Institute for Clinical and Economic Review, *NICE* National Institute for Health and Clinical Excellence, *SMC* Scottish Medicines Consortium

A data extraction form was developed based on the template first presented by Drummond et al. [4] following verification of updates needed through the targeted review (see Table 2). The data extraction form included three sections: the first relates to clinical effectiveness assessments, including items such as sample size and duration of pivotal clinical trials used to approve the therapies, and whether trials were single-arm or uncontrolled; the second section relates to the valuation of benefits for gene therapies, including the treatment of severe disease, value to caregivers, scientific spillovers, and substantial improvements in life expectancy; the third section relates to additional considerations, such as discounting or consideration of alternative payment models.

**Table 2:** Checklist for assessing gene therapies

Item	Yes	No	Notes
***Clinical Effectiveness***			
Surrogate endpoint used	□	□	Validation given?
Rare disease	□	□	Prevalence _____
Serious condition	□	□	
Single-armed trial	□	□	Matched historical cohort used?
Pediatric population	□	□	Age range _____
Reporting of adverse consequences and risks	□	□	
Size of clinical trial	_____ number of patients		
Length of clinical trial	_____ duration in months		
Extrapolation to long-term outcomes	_____ duration in months		
***Elements of Value***			*Quantification*
Severe disease	□	□	
Value to caregivers	□	□	
Insurance value	□	□	
Scientific spillovers	□	□	
Lack of alternatives	□	□	
Substantial improvement in life expectancy	□	□	
***Other Considerations***			*Notes*
Discounting			
Different discount rates explored	□	□	
Uncertainty			
Alternative payment models explored	□	□	

Source: Drummond et al., 2019^4^

The checklist has been previously used by Huygens et al. [16] in a smaller study of a single gene therapy in 3 jurisdictions. We applied it by recording whether each item was considered to be relevant in the jurisdictions concerned (Yes/No/Not available), together with illustrative quotes from the HTA reports analysed. Data extraction was performed by one researcher and a random sample of the reports (*n* = 10) was checked for accuracy of data extraction by another member of the research team.

We also conducted a more detailed analysis of the comments made in cases where a particular item was considered, by classifying the comments as ‘positive’, ‘neutral’ or ‘negative’. Positive assessments were those where the HTA body considered that the manufacturer had made a reasonable attempt at dealing with the issue, or where the HTA body recognised the importance of the item in considering the technology. Negative assessments were those where the HTA body felt that the issue had not been adequately addressed, or where it did not recognise the importance of the item in considering the technology. Neutral assessments were those where the committee made no comments either way. These assessments were made by two members of the research team, with a consensus being reached in cases where there were any differences of opinion.

The findings were discussed by a small group consisting of academic researchers, payers, patient representatives and pharmaceutical industry personnel, prior to drafting a paper for submission to a peer-reviewed journal.

## Results

### Literature review

In total 38 papers were selected for detailed review based on their title and abstract, plus 8 reports from HTA bodies and related organizations. The main issues relating to the use of evidence were as follows.

#### Clinical evidence requirements

Although the main focus of most of the papers reviewed was the economic evidence needs, several also discussed issues with the underlying clinical evidence for cell and gene therapies [4, 16, 23, 24].

##### Use of surrogate endpoints

Many cell and gene therapies are approved based on accelerated approval pathways, which allow for trials to be powered on surrogate, rather than final, endpoints. Where surrogates are used, they need to be validated, to show that they are closely linked to the final outcome of interest. This would usually be demonstrated through a meta-analysis of randomised controlled trials in the same indication [25].

However, in the case of cell and gene therapies, many of which are for rare conditions, these high standards of validation are difficult to achieve. It may be necessary to relax the requirements for validation to consider the partial exchangeability of evidence across treatment classes or other patient populations. It may also be necessary to accept that although the outcome measure used is not a true surrogate for the final outcome, it may have prognostic value for an outcome of importance to patients, carers or payers [26].

More generally on the appropriate selection of endpoints, the Institute for Clinical and Economic Review (ICER) evaluation framework for assessing single or short-term therapies (ICER-SST), has recognized the importance of patient-centric outcomes, such as daily function, chronic pain, physical activities, goal attainment, which could be relevant in many indications for cell and gene therapy, although they are rarely included in clinical trials [27].

##### Lack of comparative evidence and the use of single arm trials

Economic evaluations rely on an estimate of the relative treatment effect between the new treatment of interest and the current standard of care. However, for cell and gene therapies there is a high preponderance of single arm uncontrolled clinical studies, either because the patient population is small, or because of the practical difficulties or ethical concerns in randomization due to the potential of cure offered by the new therapy.

Therefore, it may be necessary to construct a comparison arm by using a historical or synthetic cohort. This was the approach followed in the economic evaluation of voretigene neporvovec (Luxturna) for inherited retinal disease. However, when using a non-randomized comparison it is important to adjust for any confounding factors [4]. In the absence of well-designed and conducted RCTs to establish the comparative effectiveness of treatments, epidemiologists have proposed causal inference methods rooted in counterfactual theory applied to large observational databases, that can be viewed as an attempt to emulate a randomized experiment (the target trial) that would answer the question of interest [28].

Target trial emulation is gaining traction as a two-step methodological approach, useful in situations where there is dearth of comparative evidence [29, 30]. Whilst it has been applied in several settings already, it requires large observational datasets with rich data to provide unbiased answers to the question of interest. This latter requirement makes the approach currently prohibitive to many indications of cell and gene therapies, which are developed for rare or ultra-rare conditions. Going forward, rethinking of the structure of real-world data collection, harmonization of related databases, and promotion of cross-border data sharing procedures could facilitate use of target trial emulation for the evaluation of comparative effectiveness and safety of cell and gene therapies.

##### Limited sample size in clinical studies

The problem of small patient populations for rare diseases is a feature of both randomized and non-randomized clinical studies of cell and gene therapies. One review found that 47.2% of gene therapy clinical trials enrolled fewer than 20 patients and that the median size of trial populations was 213 patients [23]. The HTA by NICE of Strimvelis, considered in the review of HTA reports (see later), was based on data from only 18 patients.

Small sample sizes greatly increase the uncertainty around clinical effect size, and also mean that any heterogeneity in the patient population is hard to analyse. With larger sample sizes heterogeneity in clinical and cost-effectiveness can be explored by defining and comparing sub-groups of the patient population, but this may not be possible with small patient numbers. In terms of subgroup data, performing post-hoc analyses without prior specifications is not appropriate, but they have been reported for some gene therapies assessed by EMA, and were found to be hypothesis generating instead of confirming [24]. An issue related to small sample size is that the study is often conducted in a single clinical centre, which can have implications on the generalizability of outcomes, because these studies are known to show larger effect estimates compared with multi-centre studies [31].

##### Immaturity of evidence and extrapolation of long-term endpoints

Another major consequence of accelerated marketing approval for cell and gene therapies is that the length of the available clinical studies may be short at the time the HTA is conducted, and hence extrapolation to long term endpoints will be required. This means that there is often considerable uncertainty about the nature of the survival curve (for the outcome of interest) for cell and gene therapies, and how this should be modelled. This issue is magnified in HTA evaluations focusing on young, compared with older, patients [32].

Some assessments of cell and gene therapies use mixture cure models, as was the case for axicabtagene ciloleucel (Yescarta), where the key assumption is that of the proportion of patients cured [33]. In the case of the evaluation of Luxturna, Huygens et al. [16] argue for an extensive use of scenario and sensitivity analyses to explore the effect of different assumptions and to characterise the uncertainty in long-term effectiveness.

#### Economic evidence requirements

Most of the papers reviewed discussed the economic evidence requirements implied by the features of the diseases treated by cell and gene therapies, or the effects of those treatments.

##### Severity of disease

Several papers noted that the conditions treated by cell and gene therapies are life threatening or seriously debilitating. There is some evidence that individuals place a higher value on health gains for people whose health is at a lower level [34]. Therefore, it is important that the nature of the disease is considered in HTAs, and the potentially higher value placed on health gains in severe disease is reflected in the analysis undertaken. In those jurisdictions that estimate the incremental cost per quality-adjusted life-year (QALY) gained from new therapies, this could involve applying a higher QALY weighting, or by raising the decision-making ‘threshold’ used to decide on whether the new technology is recommended for use [35].

##### Insurance value

Insurance value relates to the notion that individuals place a value on therapies being available in the future, even if they are unlikely to need them. It has been argued that when the treatment is for a serious disease, the insurance value is likely to be higher [36]. Some papers argue that this would apply to many of the diseases treated by cell and gene therapies [3], and methods to explore this have been suggested [37, 38]. Insurance value could be estimated by asking individuals what they would be willing to pay for an insurance premium offering them access to particular therapies, should they need them in the future. However, we are not aware of any estimates of insurance value for cell and gene therapies that have been published to date and it is unlikely that technology manufacturers have provided any estimates in submissions they have made to HTA bodies to date.

##### Lack of alternative therapies

It is often the case for rare diseases that currently no active treatment options exist. Although the definition of “unmet need” is heterogenous across countries, lack of an effective available therapeutic option is often, together with disease severity, a key qualifier for high unmet need, which constitutes a background for the value assessment of cell and gene therapies [19, 39]. Those cell and gene therapies targeting rare diseases are often compared with best supportive care. In jurisdictions using either the metrics of ‘added clinical value’ (e.g., France, Germany, and Italy), or ‘QALYs gained’ (e.g., England), the assessment should suggest a high added value if the new therapy is effective. Therefore, one might question why a treatment in situations where no treatment exists should attract additional value per se, or be provided even if it is not judged to be cost-effective. The justification given in the literature is often based on equity or ethics, because in such situations the new therapy is providing a treatment option to individuals who are currently under-served, thereby reducing health inequalities [40].

##### Step change gain in length or quality of life

Many cell and gene therapies have been described as ‘transformational’ in terms of the increases in length or quality of life they produce [41]. As in the case of comparisons with basic supportive care, effective therapies resulting in a ‘step change’ gain are likely to generate high estimates of QALYs gained. Some HTA bodies do recognise ‘step change’ innovation in their processes by using ‘modifiers’ to the decision-making incremental cost-effectiveness threshold [42]. Also, in its Highly Specialised Technologies programme, NICE allows an increase in the decision-making threshold for therapies offering patients a gain of more than 10 QALYs over their lifetime [35]. However, this is one of only a few examples of a quantification of the added benefit from step changes in length or quality of life.

Many cell and gene therapies have curative intent, although the evidence does not currently exist to show that they provide a ‘cure’ [43]. It has been argued that a cure might be more valuable than a life-long treatment of equal effectiveness for the same condition, on the grounds that continuous treatment imposes a burden on patients and continually reminds them of their health problem [44].

However, to date there are no published estimates demonstrating the value of this.

##### Option value

Although there is currently no evidence that cell and gene therapies provide a cure, many of them have a treatment effect of long duration. It has been argued in the literature that this adds value in that it provides patients the opportunity to benefit from other treatment advances in the future [36]. Option value has not yet been demonstrated in the context of gene therapy, but a recent systematic review has identified 12 estimates of option value, mostly from the field of oncology [45].

##### Value to carers and the family

The value of therapies to carers and the family, in the reduction of the emotional stress of seeing a close relative or friend suffering from a serious disease, or the time spent in providing informal care, is widely recognised but rarely quantified in HTAs [46]. Many cell and gene therapies are for diseases affecting children, where the impacts on the family may be greater, than for therapies more generally. This item links to the need to conduct economic evaluation from the societal perspective, and to pay attention to the estimation of indirect and future costs [19].

##### Scientific spillovers

When a drug with a new mechanism of action is discovered, it may facilitate the development of other therapies that will deliver benefits to future patients. Given that gene therapy is in early stages of development, it is likely that the level of scientific spillovers could be extensive. However, this has yet to be demonstrated [4]. Some HTA bodies do recognise the potential existence of scientific spillovers, and ‘new mechanism of action’ is one of the contextual considerations taken into account by ICER in the US [47].

##### Discounting

The choice of discount rate typically has a profound effect on the cost-effectiveness of cell and gene therapies, given their high up-front cost and potentially long-term benefits. Arguments have been made for a lower discount rate for gene therapies, on the grounds that the social rate of time preference might be different for investments made collectively by society, that benefit future generations [2]. However, most HTA agencies have a standard discount rate that they use for all their assessments. Nevertheless, there may be a case for undertaking a sensitivity analysis on the discount rate so that decision-makers are aware of the difference it makes.

##### Uncertainty

Although there are well-accepted methods for characterising uncertainty in economic evaluations, the main uncertainty with cell and gene therapies is the long-term durability of the clinical effect. It has been suggested that it might be useful to conduct a series of scenario analyses for long-term effect that decision-makers could contemplate [14]. A critical limitation to the economic evidence for cell and gene therapies is the reliability of opinion-based assumptions necessary for the cost-effectiveness model development when evidence is absent [18].

Uncertainty has also been referenced with respect to ancillary medical costs of administering cell and gene therapies, treating complications, providing follow-up care, and to patients and their families out-of-pocket expenses to travel to administration sites and to stay there for follow-up monitoring [24].

Overall, based on the literature review, we concluded that the issues raised in the Drummond et al. paper were still valid and representative of the current literature.

#### Evidence post-launch

Some of the long-term uncertainties can only be resolved by further data collection post-launch. Several of the papers reviewed stressed the importance of managed entry, or market access agreements for cell and gene therapies, either because of the high cost of these products and/or the uncertainty over their clinical effectiveness [4, 6]. These agreements can either be financial/utilization-based or outcomes-based. The former can include simple price cuts, price/volume agreements, price caps or staged payments. The latter can include coverage with evidence development or performance-linked reimbursement. The appropriateness of these alternative payment models depends on several factors, from the payer’s willingness and capacity to take on financial risks and administrative burden, to characteristics of the potentially indicated patient population, attributes of the therapy, or ability to assess target outcomes [48].

Outcomes-based agreements can be on the population level and dependent on the results of an ongoing prospective clinical study, such as a randomised trial, or on an ad hoc post-marketing observational study. They can also be on the patient level and dependent on a patient registry that is established as part of the agreement. The collection of evidence post-launch is critical to the outcomes-based agreements and can raise several challenges in study design, data collection and analysis. For example, one of the barriers identified for the implementation of outcome-based spread payments is reaching an agreement on financial terms while considering 12-months budget cycles and the possible violation of corresponding international accounting rules [49]. Because of these practical and administrative challenges, outcomes-based agreements have declined in popularity overall but still seem to be common for cell and gene therapies, since these therapies often pose uncertainty issues that can only been resolved by long-term data collection [13, 50].

The key question is how to collect reliable data on outcomes while still making the process manageable. Three approaches can be observed in the literature. First, data on outcomes can be obtained from clinical studies that are still ongoing at the time market access is granted. This is one of the approaches used to support the reimbursement of drugs on the Cancer Drugs Fund in the UK [51]. Secondly, new data collection in clinical practice could be initiated by undertaking ad hoc studies or by establishing patient registries [13]. This approach has been recently introduced in Germany, with the Law for More Safety in the Supply of Medicines (GSAV) in 2019 [52]. According to this law, routine clinical practice data collection can be required for orphan medicines, drugs with conditional approval, or those approved under exceptional circumstances. The aim of this data collection is to achieve a valid quantification of the added benefit, considering that in most circumstances the added benefit is not quantifiable using pivotal studies [53]. Finally, use can be made of an existing drug registry initiated for another purpose. This latter approach has been extensively used in Italy [54] and in Spain [12, 13, 55].

Finally, the literature has identified additional procedural and organizational challenges [56], from assessors’ lack of experience and preparedness to organizational differences between payers, from beneficiary turnover in multi-payer systems to lack of cross-border access which exacerbates geographical distances to be travelled by many patients [24]. These barriers are amplified in low- and middle-income countries where reliable and nationally oriented programs for HTA and adequate financial coverage of these therapies are still uncertain [57].

### Analysis of HTA reports

In total 46 HTA reports were reviewed, covering 9 cell and gene therapies, one of which (i.e., Kymriah) had indications for use in two different patient populations (i.e., relapsed or refractory diffuse large b-cell lymphoma (DLBCL), relapsed or refractory b-cell acute lymphoblastic leukaemia (ALL). Given the different timing of market access for a given technology in different jurisdictions, the list of cell and gene therapies identified was assessed by a maximum of 7 HTA bodies (e.g., Zolgensma assessed by all HTA bodies except for AEMPS) to a minimum of one single assessment (e.g., Strimvelis only assessed by NICE). None of the therapies had been assessed in all 8 jurisdictions.

#### Consideration of clinical evidence

Table 3 gives an overview of the elements of clinical evidence considered across the 46 HTA reports.

**Table 3:** Clinical evidence considerations of each of the therapies studied

Drug Name (Number of assessments)	Kymriah DLBCL (6)	Kymriah ALL (6)	Yescarta (7)	Luxturna (7)	Strimvelis (1)	Imlygic (3)	Alofisel (5)	Provenge^a^ (2)	Glybera (2)	Zolgensma (7)
Surrogate endpoint used	Yes (5)	Yes (5)	Yes (6)	Yes (6)	Yes (1)	Yes (2)	Yes (4)	No (2)	Yes (1)	Yes (2)
Rare disease	Yes (3)	Yes (3)	Yes (4)	Yes (7)		No (1)	Yes (4)	No (2)	Yes (2)	Yes (7)
Serious condition	Yes (5)	Yes (5)	Yes (6)	Yes (6)	Yes (1)	Yes (2)	Yes (4)			Yes (6)
Single-arm trial	Yes (6)	Yes (6)	Yes (7)	No (7)	Yes (1)	No (3)	No (5)	No (2)	Yes (2)	Yes (7)
Pediatric population	No (6)	Yes (5)	No (7)	Yes (2)	Yes (1)	No (3)	No (5)	No (2)	No (2)	Yes (7)
Reporting of adverse consequences and risks	Yes (6)	Yes (6)	Yes (7)	Yes (7)	Yes (1)	Yes (2)	Yes (5)	Yes (2)	Yes (1)	Yes (7)
Size of clinical trial (number of patients)	99–167	59–97	111	29–31	18	436	212–289	512	27	15–33
Length of clinical trials (months)	13.9–40.3	8.7–30.2	8.7–27.1	12–48		48	12–24	20.6–34	< 12	12–53
Extrapolation to long-term outcomes (months)	552	> 1,000	528			360	480	120		924

^a^Withdrawn from the market

The full data extraction for all the HTA reports is given in Appendix 1 (Supplementary Information). This explains in detail the nature of the consideration given by each HTA body of the different data elements and any concerns that it had.

Overall, the consideration of the key aspects of the clinical evidence by the various jurisdictions was high. Given the clinical evidence considered in HTA reports of cell and gene therapies typically relies on the pivotal clinical trials used to gain – usually accelerated – regulatory approval, there is high consistency in the consideration of clinical evidence related challenges across the different HTA bodies. For example, the use of a surrogate endpoint was discussed in 70% of the reports, the use of a single arm trial in 85% and the extrapolation to long term outcomes in 78%. The sample size of the clinical trials considered ranged from 18 (Strimvelis) to 436 (Imlygic), with a follow-up from less than 12 months (Glybera) to up to more than 4 years in the case of Zolgensma. The short duration of many of the trials motivates the frequent need of extrapolation of treatment benefit in the long term.

Taking the example of Kymriah for relapsed or refractory DLBCL, in all 6 reports analysed the use of a single arm trial and the reporting of adverse consequences and risks was discussed, together with the unanimous recognition that the therapy was not for a paediatric population. However, the fact that the therapy was for a rare disease and for a serious condition was only explicitly considered in 3 and 5 jurisdictions respectively, reflecting different judgments made by the HTA bodies of what constitutes rarity or seriousness of a disease. All agencies but G-BA considered objective response rate as the main endpoint to assess the effectiveness of the intervention, without discussing its surrogacy validity. Depending on the timing of the submission and repeated assessments, the sample size of the clinical trial considered was between 99 and 167, with a median observation time up to 40 months. A matched-adjusted indirect treatment comparison was needed to determine the comparative effectiveness of the treatment, relying on the SCHOLAR-1 cohort in DLBCL [58]. However, the payers expressed concerns due to lack of information on relevant confounders, and inclusion criteria of the cohort study identified.

**Fig. 1:**
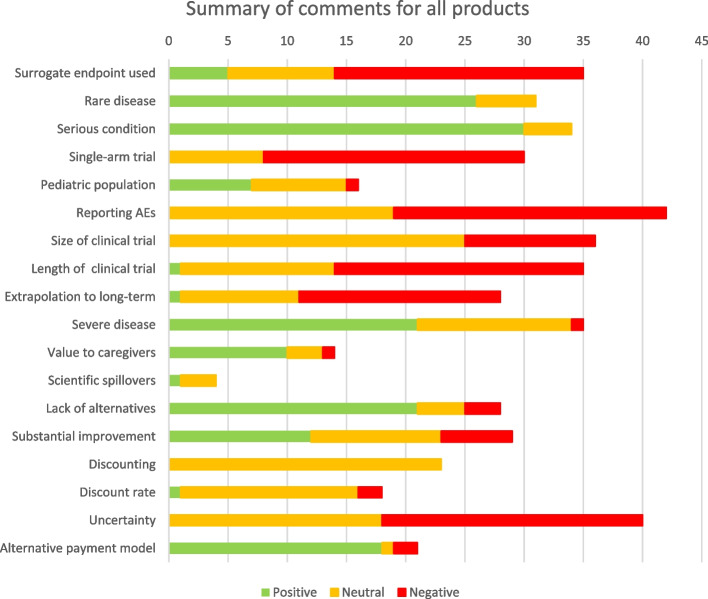
Summary of comments for all products

Although more open to judgment and interpretation, the more detailed analysis of comments indicated that the items to which the HTA bodies reacted positively were the indication that the treatment was for a rare disease and that it was a serious condition. The items to which they reacted negatively were the use of unvalidated surrogate endpoints, single arm trials without an adequately matched alternative therapy, inadequate reporting of adverse consequences and risks, the short length of follow-up in clinical trials and the methods for extrapolating to long-term outcomes (see Fig. 1). Although a direct comparison is not possible, many of these HTA body responses are consistent with those obtained by ten Ham et al. [59] in their categorisation of HTA reports on ATMPs in 3 jurisdictions into the EUnetHTA Core Model domains.

#### Consideration of economic evidence

Table 4 shows the consideration of the various elements of economic evidence in the 7 jurisdictions that had assessed onasemnogene abeparvovec (Zolgensma), one of the latest gene therapies to be assessed. The economic evidence was not applicable for the report from the G-BA since its remit is to consider only the clinical evidence. In addition, consideration of insurance value and scientific spillovers was largely lacking in all jurisdictions, as many of the HTA bodies do not consider these to be part of their remit.

**Table 4:** References to the various elements in HTA reports for onasemnogene abeparvovec (Zolgensma)

Item	NICE	ICER	CADTH	SMC	AIFA	HAS	G-BA
Severe disease	Yes	Yes	Yes	Yes	Yes	Yes	N/A
Value to caregivers	Yes	Yes	Yes	Yes	N/A	Yes	N/A
Insurance value	N/A	N/A	N/A	N/A	N/A	N/A	N/A
Scientific spillovers	Yes	N/A	N/A	N/A	N/A	N/A	N/A
Lack of alternatives	No	No	No	No	No	No	N/A
Substantial improvement in life expectancy	Yes	Yes	Yes	Yes	Yes	Yes	N/A
Discounting	Yes	Yes	N/A	Yes	Yes	Yes	N/A
Different discount rates employed	Yes	Yes	N/A	Yes	Yes	Yes	N/A
Uncertainty	Yes	Yes	Yes	Yes	Yes	Yes	Yes
Alternative payment models explored	No	N/A	No	No	Yes	N/A	N/A

N/A = information not available in the HTA report or supporting material

As in the case of the clinical evidence considerations, the details of the full data extraction are given in Appendix 1.

As compared with the clinical evidence considerations, there was much more variability in the extent to which the various elements of economic evidence were considered (see Table 5). In particular, it is notable that although in 50% of reports discounting was applied, in only 37% were different discount rates explored. Also, in only 30% of cases was the value of therapy to caregivers explored.

**Table 5:** Summary statistics on consideration of economic items

Item	Considered relevant No. (% of reports)
Severe disease	35 (76%)
Value to caregivers	14 (30%)
Insurance value	0 (0%)
Scientific spillovers	4 (9%)
Lack of alternatives	28 (61%)
Substantial improvement in life expectancy	29 (63%)
Discounting	23 (50%)
Different discount rates employed	18 (39%)
Uncertainty	40 (87%)
Alternative payment models explored	21 (46%)

The more detailed analysis of comments indicated that the items to which HTA bodies reacted positively were that the treatment was for a severe disease, that there was a lack of alternative therapies, that the treatment delivered a substantial improvement in life expectancy, and that an alternative payment model had been explored or agreed (see Fig. 1).

#### Consideration of evidence post-launch

Consideration of evidence post-launch often forms part of market entry agreements for cell and gene therapies. The existence of a market entry agreement is often mentioned in HTA reports. However the details of those agreements, the evidence demands they address and data collection plan are systematically available for population-based agreement in England.

Table 6 shows the details of the outcomes-based agreements for which publicly available data are available in France, Germany, Italy, and Spain. Details of the 4 agreements existing in England are illustrated in Table 7.

**Table 6:** Outcome-based managed entry agreements for gene therapies in France, Germany, Italy and Spain

Gene & Cell therapies	France	Germany	Italy	Spain
Strimvelis	-	-	Performance-linked-reimbursement (through Drug Registry)^a^	-
Kymriah	The Transparency Committee recommended to collect further data through the Lymphoma Academic Research Organization Registry: effectiveness (survival, remission status, disease progression) and adverse events, to be recorded at 28 days, 100 days, 6 months and every subsequent 6 months after injection^b^	Rebates to the health insurers for patients dying after treatment (time horizon for measuring survival, and the magnitude of the rebate not available in public dominion)^b^	Outcome-based staged payments. Installments: infusion, 6 months, 12 months Outcome indicator: unknown (confidentiality agreement)^a^	Outcome-based staged payments. Installments: time of treatment (52%), 18 months (48%) Outcome indicator: complete response to the treatment^b^
Yescarta			Outcome-based staged payments. Installments: 180, 270, 365 days after infusion Outcome indicator: unknown (confidentiality agreement)^a^	Outcome-based staged payments. Installments: time of treatment (52%), 18 months (48%) Outcome indicator: survival^b^
Luxturna	-	-	-	Price agreement with additional data collection^a^
Zolgensma	-	Mandatory collection of long-term RWD until 2027^a^	Outcome-based staged payments. Installments: 0, 1, 2, 3, 4 years Outcome indicator: unknown (confidentiality agreement)^a^	Price agreement with additional data collection^a^

*RWD* real-world data

Sources:

^a^ ATMP Forum [55]: Quarto Report italiano sulle Advanced Therapy Medicinal Product (https://www.atmpforum.com/report/)

^b^ Jørgensen et al. [13]; Jørgensen et al.[12]

**Table 7:** Managed access agreement in England for gene therapies

Managed Access Agreements	Kymriah DLBCL^a^	Kymriah ALL^b^	Yescarta (both indications)^c^	Zolgensma^d^
General concerns	Most plausible ICER for Kymriah compared to salvage chemotherapy is uncertain, but Kymriah has the plausible potential to satisfy the criteria for routine use if this uncertainty could be reduced	Most plausible ICER for Kymriah is higher than the threshold range and Kymriah does not meet the criteria to be a ELT	Most plausible ICER for Yescarta compared to salvage chemotherapy is uncertain. The range of ICER estimates show that Yescarta has plausible potential to be cost-effective and collecting further data on PFS, OS and IVIG use will reduce uncertainty in the evidence	- Robustness of the economic modelling: the model assumes that everyone with pre-symptomatic SMA and up to 3 copies of the SMN 2 gene would develop type 1 SMA, whereas a substantial proportion of this population would be expected to develop other types of SMA - Data that would address other uncertainties (e.g. generalizability of trial evidence) cannot be collected within a reasonable timeframe and any additional data collection would be burdensome for patients and clinicians
Uncertainties	OS: immature data that do not fully support the curative nature of Kymriah	OS: immaturity data that not fully support the curative nature of Kymriah	OS: median overall survival not been reached in pivotal trial (ZUMA-1)	
	Proportion of patients who would need treatment for B-cell aplasia with IVIG and duration of the treatment	Subsequent stem cell transplant rates	PFS and OS: timeline over which that the curves for progression-free survival and overall survival may converge is not available	
		Proportion of patients who would need treatment for B-cell aplasia with IVIG and duration of the treatment	Use of IVIG: need for IVIG in a real-world setting remains uncertain, even data from the follow-up of pivotal trial (ZUMA-1) demonstrated that IVIG was rarely used (8.3% patients)	
Data collection and sources for the post- HTA evidence	OS - Long-term data coming from the follow-up of pivotal trial (JULIET—Final Clinical Report expected on Aug 2023) - Routine population-wide datasets (NHS SACT)	OS - Long-term data coming from the follow-up of pivotal trial (ELIANA—Final Clinical Report expected on Jun 2023)	OS - Long-term data coming from the follow-up of pivotal trial (ZUMA-1) (5-year data available in Q1 2022 and confidentially reported to NICE)	Different endpoints for two cohort of patients (SMN2 and SMN3) patients - Long-term data coming from the follow-up of the Clinical Trial (SPR1NT), that will be incorporated into an evidence submission and the new economic (expected for July 2022 but not tracked on NICE's website so far) Baseline patient characteristics, including gender, age, date of diagnosis, and SMN2 copy number - NHS England Blueteq system
	IVIG - Long-term data coming from the follow-up of pivotal trial (JULIET—Final Clinical Report expected on Aug 2023) - Integrated dataset (MDSAS and NHS England’s Blueteq)	Stem cell transplant rates BMT Registry (stem cell transplant rates) (it is anticipated that there may be difficulties in linking these to national datasets, to identify patients treated with Kymiriah)	Timeline convergence of OS and PFS - Long-term data coming from the follow-up of pivotal trial (ZUMA-1) (5-year data available in Q1 2022 and confidentially reported to NICE)	
		IVIG Not specified, but the NHS England’s Blueteq is mentioned	IVIG - Long-term data coming from the follow-up of pivotal trial (JULIET—Final Clinical Report expected on Aug 2023) - Integrated dataset (MDSAS and NHS England’s Blueteq)	

*ALL* Acute Lymphoblastic Leukemia, *BMT* Bone Marrow Transplant, *DLBCL* Diffuse Large B-Cell Lymphoma, *ELT* End of Life Treatment, *ICER* Incremental Cost-Effectiveness Ratio, *IVIG* Intravenous Immunoglobulins, *MDSAS* Medical Data Solutions and Services, *OS* Overall Survival, *PFS* Progression-Free Survival, *SACT* Systemic Anti-Cancer Therapy, *SMA* Spinal Muscular Atrophy

Sources

^a^NICE. Cancer Drugs Fund Managed Access Agreement. Tisagenlecleucel for treating relapsed or refractory diffuse large B cell lymphoma after 2 or more systemic therapies [TA567] (https://www.nice.org.uk/guidance/ta567/documents/committee-papers-4)

^b^NICE. Cancer Drugs Fund Managed Access Agreement. Tisagenlecleucel for treating relapsed or refractory B-cell acute lymphoblastic leukaemia in people aged

up to 25 years [TA554]

(https://www.nice.org.uk/guidance/ta554/resources/managed-access-agreement-december-2018-pdf-6651288397)

^c^NICE. Cancer Drugs Fund Managed Access Agreement. Axicabtagene ciloleucel for treating diffuse large B cell lymphoma and primary mediastinal B cell lymphoma after 2 or more systemic therapies [TA559]

^d^NICE. Managed Access Agreement. Onasemnogene abeparvovec for pre-symptomatic 5q spinal muscular atrophy (SMA) with a bi-allelic mutation in the SMN1 gene and up to 3 copies of the SMN2 gene [HST15]

(https://www.nice.org.uk/guidance/hst15/resources/managed-access-agreement-pdf-9191290285)

The population-based agreements agreed between manufacturers and NICE/Department of Health and Social Care in England include a structured data collection protocol but, as was mentioned before, they are not normally de novo observational studies but rely on ongoing clinical studies/trials and existing NHS data sources. These are aimed at reducing uncertainty in the estimates of outcomes and cost-effectiveness. They generally do not include gathering of data on other patient-relevant endpoints unless these were already being gathered in the ongoing trials.

The publicly available data on population-based schemes in France was too limited to enable any assessment of their suitability.

The experience of Germany on routine practice data collection for the evaluation of added benefit is very recent and driven by the GSAV law. A study protocol for routine data collection and evaluations of onasemnogene abeparvovec (Zolgensma) was published in January 2022 [60]. The objective of the study is to evaluate the overall effectiveness and safety in patients with spinal muscular atrophy (SMA) treated with Zolgensma compared with Spinraza (nusinersen) based on a non-interventional, non-randomized data collection using secondary data from two registries. The data cut for the final analysis is planned for December 2026.

The individual-based agreements in Spain and Italy rely on data collected through drug registries and provide enough data on patient response to identify non-responders (for purposes of determining clawback payments) and to inform staged payments. However, the data collected through these registries are not incorporated into a wider programme for real world data collection and do not normally provide enough data to assess clinical outcomes or the impact of therapies on health-related quality of life. The role of these registries in any re-appraisal process is unknown and the data are not normally published.

## Discussion

Based on the findings of the literature review and analysis of HTA, it is possible to identify several issues in the generation and use of evidence for cell and gene therapies that require further discussion and consideration However, in discussing these issues it is important to recognise that there are differences between HTA bodies in different jurisdictions in how the clinical and economic evidence should be used. Therefore, any suggestions for improvements need to take account of these country-level differences.

To date, most HTA bodies, including those studied here, have not instituted a separate assessment programme or approach for cell and gene therapies, or advanced therapy medicinal products (ATMPs) more generally, although some have supplemental processes for rare disease treatments that are more suited to their assessment [61, 62]. Given the preference of most HTA bodies to use the same standardized approach for all health technologies, it may be more realistic to consider ways in which bodies can tackle the various challenges within their existing approach to HTA. Ultimately, any changes in how cell and gene therapies are assessed will reflect the views of the various HTA bodies and the constituencies they serve. However, a few suggestions are given below.

### Consideration of clinical evidence

In terms of establishing the validity of the surrogate endpoints, current appraisals of cell and gene therapies are unlikely to meet the highest standards (e.g., link between treatment effects on surrogate and final outcomes established based on meta-analyses of RCTs in the same indication and treatment class). However, it is still recommended that appraisals of these therapies follow a structured approach to the validation of surrogate endpoints, and justify the adoption based on the prognostic value of the biomarker rather than on the surrogacy value, on relaxed requirements of indication- and treatment- specific evidence of validation, on full or partial exchangeability of evidence across treatment classes and populations. In addition, carefully designed post-launch evidence generation programmes may answer the question of surrogate validity and effectiveness on patient-relevant endpoints, which may be the preferred approach in some jurisdictions. Therefore, it would be useful for HTA bodies and payers to agree standards for long-term patient relevant outcome collection and for validating surrogate endpoints, while acknowledging the challenges posed by small patient populations. Ideally, post-launch evidence generation should be agreed at the time of the HTA, be based on rigorous protocols, and, if possible, enforced by management agreements that make the reimbursement and price status contingent on data collection and the relevant findings.

Recruitment to conventional randomized controlled trials may be difficult due to the small target population, and may be influenced by the expectation of sizeable benefits in high unmet-need areas, that strongly enhances the attractiveness of the experimental arm over the standard of care. In addition, there may be operational hurdles, in enrolling patients outside qualified centres, and in the choice of the comparator arm. Although methods for the design and analysis of clinical trials in small populations have been proposed [63–65],they have not yet been widely accepted in the HTA community.

A high preponderance of pivotal single-arm uncontrolled trials means payers need to find, or assemble, a suitable contemporary historical cohort of patients treated according to standard practice, which can be used to determine the comparative effectiveness of the therapies. A common concern observed in the HTA reports was that ‘standard practice’ could vary across jurisdictions or change over time. Guiding principles for performing such treatment comparisons could be agreed in advance between manufacturers and decision-makers, starting from high-quality patient-level data from reliable and traceable sources; appropriate cohort selection based on matching inclusion/exclusion; suitability of real-world endpoints; fit-for-purpose analytical methodologies. The acceptability of synthetic cohorts or in silico modelling for this purpose should be established. Therefore, it would be useful to agree criteria for when a single-arm clinical study can be deemed acceptable, and guidelines for assembling a matching comparator cohort.

Due to the short timeframe of the pivotal clinical studies (typically one or two years), long-term benefit and adverse events of cell and gene therapies are not substantiated by trial data and remain largely uncertain at the time of the assessment. Therefore, there is a need to support the assumption of maintained improvement in health or treatment effect in the long-term. It is recommended that current biological knowledge on the pathophysiology of the disease complemented by additional external sources of evidence inform this step. The extrapolation of survival curves is more and more often based on mixture cure models in appraisals of cell and gene therapies, although the plausibility of presence and magnitude of a cure fraction must be discussed at length. Extensive scenario and sensitivity analyses are warranted, given that this parameter may have the largest impact on the cost-effectiveness analyses results.

Finally, quality of life measures, although important to patients, are not necessarily considered by those HTA bodies assessing added clinical value, as they are perceived as being subjective. Some countries may need to consider changing their assessment framework to encompass quality of life, although efforts to collect these data in clinical studies also need to improve, as quality of life evidence for cell and gene therapies, and rare diseases more generally, is scarce and often of poor quality [66]. It would also help if there was a requirement from regulatory or HTA bodies to undertake quality of life data collection as part of clinical trials.

### Consideration of economic evidence

In addressing the consideration of economic evidence, it is again important to recognise that HTA processes differ quite widely across countries. In those countries that assess the ‘added clinical value’ provided by therapies, economic evidence is not explicitly considered in the assessment process, although the acquisition cost of new therapies is considered as part of the price negotiation. In those countries assessing the ‘cost-effectiveness’ of new therapies, it is possible to discuss how economic value is characterised and whether the definition of ‘value’ should be broadened. It is very rare for countries to switch from one approach to HTA to the other, although France did add the consideration of cost-effectiveness as a supporting approach to its ‘clinical added value’ decision-making procedure. Therefore, in making recommendations for change, it is important to consider how these can be implemented within each of the contrasting approaches to HTA.

First, there may be arguments for expanding the HTA process to include the impacts on caregivers and the family. These are important for all therapies, but arguably particularly important in the case of therapies for very severe debilitating illnesses and those affecting children. Within the cost-effectiveness approach this suggests that impacts on caregivers’ quality of life could be assessed and possibly the additional costs imposed on families. If a broader societal perspective were adopted, costs falling on patients and families would be included [67]. In those jurisdictions adopting a narrower health care perspective, it would still be possible to present these costs separately from the costs falling on the health care system, so that decision makers can consider them.

Within the clinical added value approach, consideration of impacts on caregivers and the family could be made an explicit item of value within the ‘clinical’ value, or the composition of assessment committees could be expanded in include patient representatives, who are likely to stress these issues.

Secondly, there is broad agreement that cell and gene therapies offer the potential for transformational gains in health. Some jurisdictions following the cost-effectiveness approach do apply a ‘modifier’ to the threshold of the maximum level of incremental cost-effectiveness they are willing to accept in cases where there is a ‘step-change’ in survival and/or quality of life. There could be further examination of whether and how these modifiers are applied in practice and whether they make a difference in the assessments that are performed. It is more difficult to determine how this issue would be explored in those jurisdictions using the ‘added clinical value ‘ approach, other than comparing the decisions across a range of technology appraisals. One study comparing the assessments made by NICE in England and HAS in France did find a correlation between the level of QALYs gained and level of ASMR awarded [68]. Perhaps the notion of ‘added clinical value’ could be made more explicit, outlining the nature of the ‘added value’ that is considered relevant, both to patients and the general public, as well as to clinicians.

Thirdly, the application of discounting, within the ‘cost-effectiveness’ approach is critical in the assessment of cell and gene therapies, with their high ‘up-front’ costs and potential long-term benefits. It was noticeable in the analysis of HTA reports that where discounting was applied, the effect of differential discount rates was not explored in many cases. Given the sensitivity of the cost-effectiveness estimate of cell and gene therapies to the discount rate, a sensitivity analysis could be presented to decision-makers in all cases.

Fourthly, insurance value was not considered in any of the HTA assessments examined, probably because no evidence was presented. It is possible that this is a major element of the value of therapies for catastrophic disease and deserves more investigation. However, further research is required to determine whether the insurance value is substantial for these therapies and whether this justifies the application of a modifier within the HTA process.

Finally, a major consideration in all jurisdictions was the uncertainty surrounding cell and gene therapies. Conventional approaches for characterizing uncertainty, such as undertaking probabilistic assessment, are not very helpful when considering issues such as the long-term durability of these therapies, which is often completely unknown. Consideration could be given to the more extensive use of scenario analysis, suggested by Huygens et al. [16], or the more extensive use of outcome-based managed entry agreements, suggested by Drummond et al. [4].

### Consideration of evidence post-launch

Cell and gene therapies, and advanced therapy medicinal products in general, are often characterized by a high level of uncertainty at market launch over their (long-term) clinical and economic impact. Consequently, ad hoc post launch studies to produce real-world evidence (RWE) could be implemented, if they are needed and feasible. These studies can be linked with outcome-based managed entry agreements in the form of coverage with evidence development: initiating these agreements without an appropriate protocol and data collection may represent missed opportunities.

If post-launch data collection through an ad hoc study is not feasible, a pragmatic approach to data collection is better, e.g., the one adopted by England through Managed Access Agreements that rely on existing data collection programs managed by the industry, and existing NHS registries/administrative databases.

Individual-based agreements rely on existing drugs registries or ‘ad hoc’ data collection on ‘clinical outcome’ indicators. In many cases they do not provide a complete data set to assess the real-world impact of these therapies, but they could serve performance-linked reimbursement agreements, in which payment is made when patients respond to treatment.

In general, more transparency on outcome-based agreements and RWE collected through these agreements is needed, provided that the most sensitive data of the agreement (e.g., the actual price if the therapy is subject to a discounts) are kept confidential. Transparency would provide better feedback to those who are tasked with data collection, and would enhance the replicability of the agreements negotiated, thereby benefiting the health care community as a whole. Guidance for implementing and conducting managed entry agreements has recently been developed for rare disease treatments, which could form a basis for discussions on MEAs in cell and gene therapy [69].

## Conclusions

This research has shown that the consideration by HTA bodies of evidence relating to the particular features of cell and gene therapies is variable. In part this reflects the different approaches used in different jurisdictions. Although it is not possible to reach a complete consensus on which elements of evidence should be considered, several suggestions have been made. All jurisdictions conducting HTAs of these therapies could consider whether and how these suggestions might be incorporated within their existing approach, through strengthening deliberative decision-making or performing additional analyses.

## Supplementary Information

**Additional file 1.** 

## Data Availability

The data sets generated and analysed during the current study are included in this published article and its supplementary information files.
