# Supplementary material for: How are health technology assessment bodies responding to the assessment challenges posed by cell and gene therapy?
Source: BMC Health Serv Res. 2023 May 13;23:484. doi: 10.1186/s12913-023-09494-5 (PMC10182681; doi:10.1186/s12913-023-09494-5)
Supplement: Supplementary file 1 — Additional file 1. [file 12913_2023_9494_MOESM1_ESM.docx]

# Appendix 1

# **Detailed Analysis of HTA Reports**

This supplementary material contains the basic data extracted from the reports of HTA bodies. The first set of Tables #1 to #51, contain the data on each drug assessed by the bodies in England, Scotland, US, Canada, Italy, France and Germany. The second set of Tables #52-57, contain the data on the drugs assessed by the body in Spain.

The data can be assessed by searching for the country/agency, or the drug/indication.

# Source

England (NICE)

<https://www.nice.org.uk/guidance/ta567/history>

<https://www.nice.org.uk/Guidance/ta559/history>

<https://www.nice.org.uk/guidance/hst11/history>

<https://www.nice.org.uk/guidance/hst7/history>

<https://www.nice.org.uk/guidance/ta410/history>

<https://www.nice.org.uk/guidance/ta556/history>

<https://www.nice.org.uk/guidance/ta332/documents/prostate-cancer-metastatic-hormone-relapsed-sipuleucelt-1st-line-id573-final-appraisal-determination-document2>

<https://www.nice.org.uk/guidance/indevelopment/gid-hst10026/documents>

<https://www.nice.org.uk/guidance/gid-hst10026/documents/committee-papers>

Scotland (SMC)

<https://www.scottishmedicines.org.uk/media/4713/tisagenlecleucel-kymriah-final-august-2019-amended-3919-for-website.pdf>

<https://www.scottishmedicines.org.uk/media/4803/axicabtagene-ciloleucel-yescarta-resub-final-sept-2019-for-website.pdf>

<https://www.scottishmedicines.org.uk/media/5066/umar-voretigene-luxturna-final-november-2019-for-website.pdf>

<https://www.scottishmedicines.org.uk/media/4532/darvadstrocel-alofisel-final-dec-2018-for-website.pdf>

<https://www.scottishmedicines.org.uk/media/5813/onasemnogene-abeparvovec-zolgensma-final-feb-2021-amended-010321docx-for-website.pdf>

United States (ICER)

<http://icerorg.wpengine.com/wp-content/uploads/2020/10/ICER_CAR_T_Final_Evidence_Report_032318.pdf>

<http://icerorg.wpengine.com/wp-content/uploads/2020/10/MWCEPAC_VORETIGENE_FINAL_EVIDENCE_REPORT_02142018.pdf>

<https://icer.org/wp-content/uploads/2020/10/ICER_SMA_Final_Evidence_Report_110220.pdf>

Canada (CADTH)

<https://www.cadth.ca/sites/default/files/cdr/complete/SG0643%20Luxturna%20-%20CDEC%20Final%20Recommendation%20November%2016%2C%202020_for%20posting.pdf>

<https://www.cadth.ca/sites/default/files/cdr/complete/SG0649%20Zolgensma%20-%20CDEC%20Final%20Recommendation%20March%2026%2C%202021%20for%20posting.pdf>

Italy (AIFA)

<https://www.aifa.gov.it/documents/20142/1028586/Kymriah_Report_Tecnico_03.12.2019.pdf/944583eb-ac64-b6d3-1e1f-8fbb49bb5125>

https://www.aifa.gov.it/documents/20142/966686/KYMRIAH_13729_DLBCL_v1.0.pdf

<https://www.aifa.gov.it/documents/20142/966686/KYMRIAH_13729_ALL_v1.0.pdf>

https://www.aifa.gov.it/documents/20142/1028586/Yescarta_Report_Tecnico_12.06.2020.pdf

https://www.aifa.gov.it/documents/20142/1100047/YESCARTA_13734_DLBCL_PMBCL_INNOV_v1.0.pdf

<https://www.aifa.gov.it/documents/20142/1504529/117_Zolgensma_scheda_innovatività_GRADE.pdf>

<https://www.aifa.gov.it/documents/20142/1028586/Zolgensma_Report_Tecnico_12.07.2021.pdf>

France (HAS)

https://www.has-sante.fr/upload/docs/application/pdf/2018-12/kymriah_ldgcb_pic_ins_avis3_ct17238.pdf

https://www.has-sante.fr/upload/docs/application/pdf/2019-03/kymriah_ldgcb_15012019_avis_efficience.pdf

<https://www.has-sante.fr/upload/docs/evamed/CT-19042_KYMRIAH_LDGCB_PIC_REEV_AvisDef_CT19042.pdf>

https://www.has-sante.fr/upload/docs/application/pdf/2018-12/kymriah_lal_pic_ins_avis3_ct17202.pdf

https://www.has-sante.fr/upload/docs/application/pdf/2019-03/kymriah_lal_15012019_avis_efficience.pdf

<https://www.has-sante.fr/upload/docs/evamed/CT-19040_KYMRIAH_LAL_B_PIC_REEV_AvisDef_CT19040.pdf>

https://www.has-sante.fr/upload/docs/application/pdf/2018-12/yescarta_pic_ins_avis3_ct17214.pdf

https://www.has-sante.fr/upload/docs/application/pdf/2019-04/yescarta_20190225_avis_efficience.pdf

<https://www.has-sante.fr/upload/docs/evamed/CT-19018_YESCARTA_PIC_REEV_AvisDef_CT19018.pdf>

https://www.has-sante.fr/upload/docs/evamed/CT-17535_LUXTURNA_PIC_INS_Avis2_CT17535.pdf

<https://www.has-sante.fr/upload/docs/application/pdf/2019-09/luxturna_14052019_avis_efficience.pdf>

<https://www.has-sante.fr/upload/docs/application/pdf/2019-02/alofisel_pic_ins_avis3_ct17069.pdf>

<https://www.has-sante.fr/upload/docs/evamed/CT-14224_GLYBERA_PIC_INS_Avis3_CT14224.pdf>

<https://www.has-sante.fr/upload/docs/evamed/CT-18743_ZOLGENSMA_PIC_INS_AvisDef_CT18743.pdf>

<https://www.has-sante.fr/upload/docs/application/pdf/2021-03/zolgensma_15122020_avis_economique.pdf>

Germany (G-B)

Kymriah DLBCL

[https://www.g-ba.de/bewertungsverfahren/nutzenbewertung/532/#beschluesse](https://www.g-ba.de/bewertungsverfahren/nutzenbewertung/532/#nutzenbewertung)

[https://www.g-ba.de/bewertungsverfahren/nutzenbewertung/385/#beschluesse](https://www.g-ba.de/bewertungsverfahren/nutzenbewertung/385/#nutzenbewertung)

Kymriah ALL

<https://www.g-ba.de/bewertungsverfahren/nutzenbewertung/533/#beschluesse>

<https://www.g-ba.de/bewertungsverfahren/nutzenbewertung/386/#beschluesse>

Yescarta PMBCL

[https://www.g-ba.de/bewertungsverfahren/nutzenbewertung/406/#beschluesse](https://www.g-ba.de/bewertungsverfahren/nutzenbewertung/406/#nutzenbewertung)

Yescarta DLBCL

<https://www.g-ba.de/bewertungsverfahren/nutzenbewertung/408/#beschluesse>

Luxturna

<https://www.g-ba.de/bewertungsverfahren/nutzenbewertung/454/#beschluesse>

Strimvelis

not assessed

Imlygic

<https://www.g-ba.de/bewertungsverfahren/nutzenbewertung/243/#beschluesse>

Alofisel

<https://www.g-ba.de/bewertungsverfahren/nutzenbewertung/366/#beschluesse>

Provenge

<https://www.g-ba.de/bewertungsverfahren/nutzenbewertung/143/#beschluesse>

Glybera

<https://www.g-ba.de/bewertungsverfahren/nutzenbewertung/146/#beschluesse>

Zolgensma

<https://www.g-ba.de/bewertungsverfahren/nutzenbewertung/689/#beschluesse>

[https://www.g-ba.de/bewertungsverfahren/nutzenbewertung/561/#beschluesse](https://www.g-ba.de/bewertungsverfahren/nutzenbewertung/561/#nutzenbewertung)

*Further ATMP’s assessed (but not added in the tables):*

*Libmeldy*

<https://www.g-ba.de/bewertungsverfahren/nutzenbewertung/684/#beschluesse>

*Tecartus*

<https://www.g-ba.de/bewertungsverfahren/nutzenbewertung/657/#beschluesse>

*Zynteglo*

<https://www.g-ba.de/bewertungsverfahren/nutzenbewertung/506/#beschluesse> )

For details of Spanish reports, see page 94 onwards

# Results

**Table 1 Assessment status**

|  | **NICE** | **ICER** | **CADTH** | **SMC** | **AIFA** | **HAS** | **G-BA** |
| --- | --- | --- | --- | --- | --- | --- | --- |
| Kymriah DLBCL | Assessed |  |  | Assessed | Assessed | Assessed | Assessed |
| Kymriah ALL | Assessed | Assessed |  |  | Assessed | Assessed | Assessed |
| Yescarta | Assessed | Assessed |  | Assessed | Assessed | Assessed | Assessed |
| Luxturna | Assessed | Assessed | Assessed | Assessed |  | Assessed | Assessed |
| Strimvelis | Assessed |  |  |  |  |  |  |
| Imlygic | Assessed |  |  |  |  |  | Assessed |
| Alofisel | Assessed |  |  | Assessed |  | Assessed* | Assessed |
| Provenge | Assessed |  |  |  |  |  | Assessed |
| Glybera |  |  |  |  |  | Assessed* | Assessed |
| Zolgensma | Assessed | Assessed | Assessed | Assessed | Assessed | Assessed | Assessed |

*Avis d’efficience not available

**Table 2 Summary of the checklist for assessing gene therapies on tisagenlecleucel-T (Kymriah) for treating relapsed or refractory diffuse large b-cell lymphoma**

|  | NICE | ICER | CADTH | SMC | AIFA | HAS | G-BA |
| --- | --- | --- | --- | --- | --- | --- | --- |
| Surrogate endpoint used | Yes |  |  | Yes | Yes | Yes | No |
| Rare disease | N/A |  |  | Yes | N/A | Yes | Yes |
| Serious condition | Yes |  |  | Yes | Yes | Yes | N/A |
| Single-arm trial | Yes |  |  | Yes | Yes | Yes | Yes |
| Pediatric population | No |  |  | No | No | No | No |
| Reporting of adverse consequences and risks | Yes |  |  | Yes | Yes | Yes | Yes |
| Size of clinical trial (number of patients) | 111 |  |  | 99 | N/A | 115 actually treated, 165 ITT population;  167 ITT in 2020 | 167 |
| Length of clinical trial (months) | 29 |  |  | N/A | Median follow-up: 13.9  Maximum follow-up: 39 | 40.3 (median follow-up for response rate in 2021) | 32.6 |
| Extrapolation to long-term outcomes  (months) | 552 |  |  | 552 | N/A | 120 | N/A |
| Severe disease | Yes |  |  | Yes | Yes | Yes | N/A |
| Value to caregivers | N/A |  |  | Yes | N/A | N/A | N/A |
| Insurance value | N/A |  |  | N/A | N/A | N/A | N/A |
| Scientific spillovers | N/A |  |  | N/A | N/A | N/A | N/A |
| Lack of alternatives | No |  |  | Yes | Yes | Yes | N/A |
| Substantial improvement in life expectancy | Yes |  |  | Yes | Yes | No | No |
| Discounting | Yes |  |  | N/A | Yes | Yes | N/A |
| Different discount rates explored | Yes |  |  | N/A | No | Yes | N/A |
| Uncertainty | Yes |  |  | Yes | Yes | Yes | Yes |
| Alternative payment models explored | Yes |  |  | N/A | Yes | N/A | N/A |

**Table 3 Summary of the checklist for assessing gene therapies on tisagenlecleucel (Kymriah) for treating relapsed or refractory b-cell acute lymphoblastic leukaemia in people aged up to 25 years**

|  | NICE | ICER | CADTH | SMC | AIFA | HAS | G-BA |
| --- | --- | --- | --- | --- | --- | --- | --- |
| Surrogate endpoint used | Yes | Yes |  |  | Yes | Yes | No |
| Rare disease | Yes | N/A |  |  | N/A | Yes | Yes |
| Serious condition | Yes | Yes |  |  | Yes | Yes | N/A |
| Single-arm trial | Yes | Yes |  |  | Yes | Yes | Yes |
| Pediatric population | Yes | Yes |  |  | Yes | Yes | Yes |
| Reporting of adverse consequences and risks | Yes | Yes |  |  | Yes | Yes | Yes |
| Size of clinical trial (number of patients) | 58* | 55, 29, 75 |  |  | N/A | ELIANA: 97 included (79 treated)  ENSIGN: 73 included (58 treated). In 2021 75 included (64 treated) | ELIANA: 97  ENSIGN: 75 |
| Length of clinical trial (months) | N/A | 8.7 (median follow-up**) |  |  | 54.8 (maximum follow-up) | ELIANA: 8.4 (mean follow-up)  ENSIGN: 6  In 2021:  ELIANA: 30.2 (median follow-up for OS)  ENSIGN: 15.1 (median follow-up for OS) | ELIANA: 24.9  ENSIGN: 13.6 |
| Extrapolation to long-term outcomes  (months) | 1,056 | N/A |  |  | N/A | 120 (10 years) | N/A |
| Severe disease | Yes | Yes |  |  | Yes | Yes | N/A |
| Value to caregivers | Yes | N/A |  |  | N/A | N/A | N/A |
| Insurance value | N/A | N/A |  |  | N/A | N/A | N/A |
| Scientific spillovers | Yes | N/A |  |  | N/A | N/A | N/A |
| Lack of alternatives | Yes | Yes |  |  | Yes | No | N/A |
| Substantial improvement in life expectancy | Yes | Yes |  |  | Yes | Yes | No |
| Discounting | Yes | Yes |  |  | Yes | Yes | N/A |
| Different discount rates explored | Yes | No |  |  | N/A | Yes | N/A |
| Uncertainty | Yes | Yes |  |  | Yes | Yes | Yes |
| Alternative payment models explored | Yes | Yes |  |  | Yes | N/A | N/A |

*the manufacturer accepted to report the size of only one clinical trial, the others remained confidential.

**in one of the clinical studies (ELIANA)

**Table 4 Summary of the checklist for assessing gene therapies on axicabtagene ciloleucel (Yescarta) for treating diffuse large b-cell lymphoma and primary mediastinal b-cell lymphoma after 2 or more systemic therapies**

|  | NICE | ICER | CADTH | SMC | AIFA | HAS | G-BA |
| --- | --- | --- | --- | --- | --- | --- | --- |
| Surrogate endpoint used | Yes | Yes |  | Yes | Yes | Yes | No |
| Rare disease | N/A | N/A |  | Yes | Yes | Yes | Yes |
| Serious condition | Yes | Yes |  | Yes | Yes | Yes | N/A |
| Single-arm trial | Yes | Yes |  | Yes | Yes | Yes | Yes |
| Pediatric population | No | No |  | No | No | No | No |
| Reporting of adverse consequences and risks | Yes | Yes |  | Yes | Yes | Yes | Yes |
| Size of clinical trial (number of patients) | 108 | 22, 101 |  | 101 | 108 | 111 ITT, 101 received administration in 2017 | 111 |
| Length of clinical trial (months) | 15.4 | 15.4 |  | 27.1 | 27.1 (median follow-up) | 8.7 (median follow-up in 2017)  27.1 (median follow-up in 2020) | 23.5 |
| Extrapolation to long-term outcomes  (months) | 528 | N/A |  | N/A | 504 (42 years, lifetime) | 408 (32 years, lifetime) | N/A |
| Severe disease | Yes | Yes |  | Yes | N/A | Yes | N/A |
| Value to caregivers | N/A | N/A |  | Yes | N/A | N/A | N/A |
| Insurance value | N/A | N/A |  | N/A | N/A | N/A | N/A |
| Scientific spillovers | N/A | N/A |  | N/A | N/A | N/A | N/A |
| Lack of alternatives | Yes | Yes |  | Yes | Yes | Yes | N/A |
| Substantial improvement in life expectancy | Yes | Yes |  | Yes | Yes | Yes | No |
| Discounting | Yes | Yes |  | N/A | Yes | Yes | N/A |
| Different discount rates explored | Yes | No |  | N/A | Yes | Yes | N/A |
| Uncertainty | Yes | Yes |  | Yes | Yes | Yes | Yes |
| Alternative payment models explored | Yes | Yes |  | N/A | Yes | N/A | N/A |

**Table 5 Summary of the checklist for assessing gene therapies on voretigene neparvovec (Luxturna) for treating inherited retinal dystrophies caused by rpe65 gene mutations**

|  | NICE | ICER | CADTH | SMC | AIFA | HAS | G-BA |
| --- | --- | --- | --- | --- | --- | --- | --- |
| Surrogate endpoint used | Yes | Yes | Yes | Yes |  | Yes | No |
| Rare disease | Yes | Yes | Yes | Yes |  | Yes | Yes |
| Serious condition | Yes | Yes | Yes | Yes |  | Yes | N/A |
| Single-arm trial | No | No | No | No |  | No | No |
| Pediatric population | N/A* | N/A* | N/A* | N/A* |  | Yes | Yes |
| Reporting of adverse consequences and risks | Yes | Yes | Yes | Yes |  | Yes | Yes |
| Size of clinical trial (number of patients) | 29 | 31 | 31 | 31 |  | 31 | 31 |
| Length of clinical trial (months) | 48 | 36 (48 for a limited set of outcomes) | 12 | up to 48 |  | At least 24 (2 patients had a 36 -month follow-up and 4 patients had a 48-month follow-up) | 12 |
| Extrapolation to long-term outcomes  (months) | N/A | N/A | N/A | N/A |  | Lifetime (patients enter the model at 3 years and can live up to 100 years) | N/A |
| Severe disease | Yes | Yes | Yes | Yes |  | Yes | N/A |
| Value to caregivers | Yes | Yes | N/A | Yes |  | N/A | N/A |
| Insurance value | N/A | N/A | N/A | N/A |  | N/A | N/A |
| Scientific spillovers | Yes | Yes | N/A | N/A |  | N/A | N/A |
| Lack of alternatives | Yes | Yes | Yes | Yes |  | Yes | N/A |
| Substantial improvement in life expectancy | N/A | N/A | N/A | N/A |  | N/A | No |
| Discounting | Yes | Yes | N/A | N/A |  | Yes | N/A |
| Different discount rates explored | Yes | No | N/A | N/A |  | Yes | N/A |
| Uncertainty | Yes | Yes | Yes | Yes |  | Yes | Yes |
| Alternative payment models explored | No | No | Yes | N/A |  | N/A | N/A |

*The condition is not pediatric per se; however, onset tends to happen at a young age.

**Table 6 Summary of the checklist for assessing gene therapies on Strimvelis for treating severe combined immunodeficiency caused by**

**adenosine deaminase deficiency**

|  | NICE | ICER | CADTH | SMC | AIFA | HAS | G-BA |
| --- | --- | --- | --- | --- | --- | --- | --- |
| Surrogate endpoint used | Yes |  |  |  |  |  |  |
| Rare disease | N/A |  |  |  |  |  |  |
| Serious condition | Yes |  |  |  |  |  |  |
| Single-arm trial | Yes |  |  |  |  |  |  |
| Pediatric population | Yes |  |  |  |  |  |  |
| Reporting of adverse consequences and risks | Yes |  |  |  |  |  |  |
| Size of clinical trial (number of patients) | 18 |  |  |  |  |  |  |
| Length of clinical trial (months) | N/A |  |  |  |  |  |  |
| Extrapolation to long-term outcomes  (months) | N/A |  |  |  |  |  |  |
| Severe disease | Yes |  |  |  |  |  |  |
| Value to caregivers | Yes |  |  |  |  |  |  |
| Insurance value | N/A |  |  |  |  |  |  |
| Scientific spillovers | N/A |  |  |  |  |  |  |
| Lack of alternatives | N/A |  |  |  |  |  |  |
| Substantial improvement in life expectancy | Yes |  |  |  |  |  |  |
| Discounting | Yes |  |  |  |  |  |  |
| Different discount rates explored | Yes |  |  |  |  |  |  |
| Uncertainty | Yes |  |  |  |  |  |  |
| Alternative payment models explored | No |  |  |  |  |  |  |

**Table 7 Summary of the checklist for assessing gene therapies on talimogene laherparepvec (T-VEC) (Imlygic) for treating unresectable metastatic melanoma**

|  | NICE | ICER | CADTH | SMC | AIFA | HAS | G-BA |
| --- | --- | --- | --- | --- | --- | --- | --- |
| Surrogate endpoint used | Yes |  |  |  |  |  | No |
| Rare disease | N/A |  |  |  |  |  | No |
| Serious condition | Yes |  |  |  |  |  | N/A |
| Single-arm trial | No |  |  |  |  |  | No |
| Pediatric population | No |  |  |  |  |  | No |
| Reporting of adverse consequences and risks | Yes |  |  |  |  |  | No |
| Size of clinical trial (number of patients) | 436 |  |  |  |  |  | N/A |
| Length of clinical trial (months) | 48 |  |  |  |  |  | No |
| Extrapolation to long-term outcomes  (months) | 360 |  |  |  |  |  | N/A |
| Severe disease | Yes |  |  |  |  |  | N/A |
| Value to caregivers | N/A |  |  |  |  |  | N/A |
| Insurance value | N/A |  |  |  |  |  | N/A |
| Scientific spillovers | N/A |  |  |  |  |  | N/A |
| Lack of alternatives | Yes |  |  |  |  |  | N/A |
| Substantial improvement in life expectancy | Yes |  |  |  |  |  | No |
| Discounting | Yes |  |  |  |  |  | N/A |
| Different discount rates explored | Yes |  |  |  |  |  | N/A |
| Uncertainty | Yes |  |  |  |  |  | Yes |
| Alternative payment models explored | No |  |  |  |  |  | N/A |

**Table 8** **Summary of the checklist for assessing gene therapies on darvadstrocel (Alofisel) for treating perianal fistula in Crohn’s disease**

|  | NICE | ICER | CADTH | SMC | AIFA | HAS | G-BA |
| --- | --- | --- | --- | --- | --- | --- | --- |
| Surrogate endpoint used | Yes |  |  | Yes |  | Yes | No |
| Rare disease | Yes |  |  | Yes |  | Yes | Yes |
| Serious condition | Yes |  |  | Yes |  | Yes | N/A |
| Single-arm trial | No |  |  | No |  | No | No |
| Pediatric population | No |  |  | No |  | No | No |
| Reporting of adverse consequences and risks | Yes |  |  | Yes |  | Yes | Yes |
| Size of clinical trial (number of patients) | 289 |  |  | 212 |  | 205 (212 ITT) | 212 |
| Length of clinical trial (months) | 22 |  |  | 12, limited data on 24 |  | 24 | 12 |
| Extrapolation to long-term outcomes  (months) | 480 |  |  | 480 |  | N/A | N/A |
| Severe disease | Yes |  |  | Yes |  | Yes | N/A |
| Value to caregivers | Yes |  |  | Yes |  | N/A | N/A |
| Insurance value | No |  |  | N/A |  | N/A | N/A |
| Scientific spillovers | No |  |  | N/A |  | N/A | N/A |
| Lack of alternatives | Yes |  |  | Yes |  | No | N/A |
| Substantial improvement in life expectancy | No |  |  | No |  | No | No |
| Discounting | Yes |  |  | N/A |  | N/A | N/A |
| Different discount rates explored | Yes |  |  | N/A |  | N/A | N/A |
| Uncertainty | Yes |  |  | Yes |  | N/A | Yes |
| Alternative payment models explored | N/A |  |  | N/A |  | N/A | N/A |

**Table 9 Summary of the checklist for assessing gene therapies on sipuleucel-T (Provenge) for treating asymptomatic or minimally symptomatic metastatic hormone-relapsed prostate cancer**

|  | NICE | ICER | CADTH | SMC | AIFA | HAS | G-BA |
| --- | --- | --- | --- | --- | --- | --- | --- |
| Surrogate endpoint used | No |  |  |  |  |  | No |
| Rare disease | N/A |  |  |  |  |  | No |
| Serious condition | N/A |  |  |  |  |  | N/A |
| Single-arm trial | No |  |  |  |  |  | No |
| Pediatric population | No |  |  |  |  |  | No |
| Reporting of adverse consequences and risks | Yes |  |  |  |  |  | Yes |
| Size of clinical trial (number of patients) | 512 |  |  |  |  |  | IMPACT: 512  D9901: 127  D9902A: 98 |
| Length of clinical trial (months) | 34 (median follow-up) |  |  |  |  |  | IMPACT: 20.6 (intervention) / 19.3 (placebo)  D9901: not provided  D9902A: not provided |
| Extrapolation to long-term outcomes  (months) | 120 |  |  |  |  |  | N/A |
| Severe disease | N/A |  |  |  |  |  | N/A |
| Value to caregivers | N/A |  |  |  |  |  | N/A |
| Insurance value | No |  |  |  |  |  | N/A |
| Scientific spillovers | No |  |  |  |  |  | N/A |
| Lack of alternatives | N/A |  |  |  |  |  | N/A |
| Substantial improvement in life expectancy | N/A |  |  |  |  |  | No |
| Discounting | Yes |  |  |  |  |  | N/A |
| Different discount rates explored | N/A |  |  |  |  |  | N/A |
| Uncertainty | Yes |  |  |  |  |  | Yes |
| Alternative payment models explored | N/A |  |  |  |  |  | N/A |

**Table 10 Summary of the checklist for assessing gene therapies on alipogene tiparvovec (Glybera) for treating adults with lipoprotein lipase deficiency who have severe or multiple attacks of pancreatitis (inflammation of the pancreas) despite maintaining a low-fat diet**

|  | NICE | ICER | CADTH | SMC | AIFA | HAS | G-BA |
| --- | --- | --- | --- | --- | --- | --- | --- |
| Surrogate endpoint used |  |  |  |  |  | Yes | No |
| Rare disease |  |  |  |  |  | Yes | Yes |
| Serious condition |  |  |  |  |  | N/A | N/A |
| Single-arm trial |  |  |  |  |  | Yes | Yes |
| Pediatric population |  |  |  |  |  | No | No |
| Reporting of adverse consequences and risks |  |  |  |  |  | Yes | No |
| Size of clinical trial (number of patients) |  |  |  |  |  | 27 | N/A |
| Length of clinical trial (months) |  |  |  |  |  | <12 | N/A |
| Extrapolation to long-term outcomes  (months) |  |  |  |  |  | N/A | N/A |
| Severe disease |  |  |  |  |  | Yes | N/A |
| Value to caregivers |  |  |  |  |  | N/A | N/A |
| Insurance value |  |  |  |  |  | N/A | N/A |
| Scientific spillovers |  |  |  |  |  | N/A | N/A |
| Lack of alternatives |  |  |  |  |  | Yes | N/A |
| Substantial improvement in life expectancy |  |  |  |  |  | No | No |
| Discounting |  |  |  |  |  | N/A | N/A |
| Different discount rates explored |  |  |  |  |  | N/A | N/A |
| Uncertainty |  |  |  |  |  | N/A | Yes |
| Alternative payment models explored |  |  |  |  |  | N/A | N/A |

**Table 11 Summary of the checklist for assessing gene therapies on onasemnogene abeparvovec (Zolgensma) for treating spinal muscular atrophy**

|  | NICE | ICER | CADTH | SMC | AIFA | HAS | G-BA |
| --- | --- | --- | --- | --- | --- | --- | --- |
| Surrogate endpoint used | Yes | No | No | Yes | No | No | No |
| Rare disease | Yes | Yes | Yes | Yes | Yes | Yes | Yes |
| Serious condition | Yes | Yes | Yes | Yes | Yes | Yes | N/A |
| Single-arm trial | Yes | Yes | Yes | Yes | Yes | Yes | Yes |
| Pediatric population | Yes | Yes | Yes | Yes | Yes | Yes | Yes |
| Reporting of adverse consequences and risks | Yes | Yes | Yes | Yes | Yes | Yes | Yes |
| Size of clinical trial (number of patients) | 15 | 15 | 22 (STR1VE-US), 30 (SPR1NT) | 22 (STR1VE US)  15 (START)  13 (LT-001)  29 (SPR1NT) | 22 (STR1VE-US) | 22 (STR1VE US)  15 (START) | 15 (START)  33 (STR1VE-EU)  22 (STR1VE-US) |
| Length of clinical trial (months) | 24 | 39 | 18 | 18 (STR1VE US)  24 (START)  53 (4.4 years LT-001) | N/A | 24 | 12-25 |
| Extrapolation to long-term outcomes  (months) | Lifetime horizon | Lifetime horizon | 924 (77 years) | Lifetime horizon | Lifetime horizon | 120 | N/A |
| Severe disease | Yes | Yes | Yes | Yes | Yes | Yes | N/A |
| Value to caregivers | Yes | Yes | Yes | Yes | N/A | Yes | N/A |
| Insurance value | N/A | N/A | N/A | N/A | N/A | N/A | N/A |
| Scientific spillovers | Yes | N/A | N/A | N/A | N/A | N/A | N/A |
| Lack of alternatives | No | No | No | No | No | No | N/A |
| Substantial improvement in life expectancy | Yes | Yes | Yes | Yes | Yes | Yes | No |
| Discounting | Yes | Yes | N/A | Yes | Yes | Yes | N/A |
| Different discount rates explored | Yes | Yes | N/A | Yes | Yes | Yes | N/A |
| Uncertainty | Yes | Yes | Yes | Yes | Yes | Yes | Yes |
| Alternative payment models explored | N/A | N/A | No | No | Yes | N/A | N/A |

**Table 12 Checklist for assessing gene therapies. Drug: tisagenlecleucel-T (Kymriah) for treating relapsed or refractory diffuse large b-cell lymphoma. Setting: England (NICE)**

| Item | Yes | No | Notes |
| --- | --- | --- | --- |
| Surrogate endpoint used | X |  | **Validation given?** N/A  OS data available; however, OS was not the primary endpoint. The authority expressed concerns on maturity. |
| Rare disease | N/A | | **Prevalence**: N/A  The manufacturer reported data on incidence only. |
| Serious condition | X |  | Seriousness of the condition is reported by manufacturer, confirmed by patient organizations, and not questioned by the authority. |
| Single-arm trial | X |  | **Matched historical cohort used?** Yes |
| Pediatric population |  | X | **Age range**: “adults”; mean age at diagnosis is 70 years of age. |
| Reporting of adverse consequences and risks | X |  | Adverse events reported for JULIET single-arm phase 2 trial, safety was analyzed across all patients in Schuste 2017 case series study. |
| Size of clinical trial | **Number of patients**: 111  (JULIET trial) | | Schuster 2017 case series consists of 14 patients; therefore, the total sample size of the pooled analysis is 125. |
| Length of clinical trial | **Duration in months**: 29 (JULIET trial) | | Schuster 2017 case series lasted 29 months, median follow-up was 28.6 months. JULIET trial is still ongoing; it is expected to be terminated in 2023. |
| Extrapolation to long-term outcomes | **Duration in months**: 552 (46 years) | | The authority expressed concerns on methods and assumptions for extrapolation. |
|  | **Yes** | **No** | **Quantification** |
| Severe disease | X |  | Arguments provided by manufacturer (progression rate and prognosis compared to other NHL) and patient organizations (impact on physical conditions and quality of life). |
| Value to caregivers | N/A | | N/A |
| Insurance value | N/A | | N/A |
| Scientific spillovers | N/A | | N/A |
| Lack of alternatives |  | X | Although alternatives do exist, manufacturer and patient organizations claim the unmet need for more tolerable and more effective treatments |
| Substantial improvement in life expectancy | X |  | The company claims the treatment is potentially curative; the authority showed concerns on how this feature was incorporated in the cost-effectiveness model. |
|  | **Yes** | **No** | **Notes** |
| Discounting | X |  | 3.50% per annum discount rate for both costs and benefits |
| Different discount rates explored | X |  | In deterministic sensitivity analysis. The authority expressed concerns on the lowest value chosen. |
| Uncertainty | X |  | PSA, DSA, scenario analysis. The authority expressed concerns on characterization of uncertainty. |
| Alternative payment models explored | X |  | Managed entry agreement with evidence generation requirements. No explicit information on payment scheme. |

**Table 13 Checklist for assessing gene therapies. Drug: tisagenlecleucel-T (Kymriah) for treating relapsed or refractory diffuse large b-cell lymphoma. Setting: Scotland (SMC)**

| Item | Yes | No | Notes |
| --- | --- | --- | --- |
| Surrogate endpoint used | X |  | **Validation given?** N/A  Objective response rate was the primary endpoint, progression-free survival and overall survival are among the secondary endpoints. |
| Rare disease | X |  | **Prevalence**: N/A  The treatment met the SMC ultra-orphan criterion |
| Serious condition | X |  | Literature evidence of poor outcomes in relapsed or progressed patients (median OS of 6.3 months, 20% survival rate at two years). The authority recognized high unmet need. |
| Single-arm trial | X |  | **Matched historical cohort used?** The manufacturer performed a naïve, unadjusted, indirect comparison of overall survival between pivotal clinical study (JULIET) data and data from Haematological Malignancy Research Network. The authority expressed concerns on data used as comparator and requested additional analyses using CORAL extension studies and SCHOLAR-1 registry data. |
| Pediatric population |  | X | **Age range**: N/A |
| Reporting of adverse consequences and risks | X |  | The authority expressed concerns on lack of longer term safety data |
| Size of clinical trial | **Number of patients**: 99 | | At the time of the primary analysis (March 2017), 147 patients had been enrolled and 99 had been treated with tisagenlecleucel |
| Length of clinical trial | **Duration in months**: N/A | | Median duration of follow-up not reported. The authority expressed concerns on the impact of short median duration of follow-up and high censoring rate on clinical benefits estimates. |
| Extrapolation to long-term outcomes | **Duration in months**: 552 | | The economic analysis applied a spline model with three knots and a spline model with one knot to the observed data for PFS and OS respectively. The authority expressed concerns on parametric models for treatment OS and the fact that no cure assumption was used in the modelling of OS for the comparator (salvage chemotherapy). |
|  | **Yes** | **No** | **Quantification** |
| Severe disease | X |  | The treatment met the SMC end-of-life criteria. The authority recognized very poor prognosis for progressed or relapsed patients and the limited availability of effective treatments to control DLBCL symptoms. |
| Value to caregivers | X |  | The single administration was considered an advantage over cycles of chemotherapy to patients and their families according to patient organizations. |
| Insurance value | N/A | | N/A |
| Scientific spillovers | N/A | | N/A |
| Lack of alternatives | X |  | No standard treatment for relapsed or progressed patients and treatment options are currently limited to salvage or palliative chemotherapy, stem cell transplant for a very small number of suitable patients and enrolment in a clinical trial. The authority recognized high unmet need. |
| Substantial improvement in life expectancy | X |  | ORR 52%, 40% complete response rate, 11.7 months median OS. The authority recognized the potential for the treatment to provide durable complete response. |
|  | **Yes** | **No** | **Notes** |
| Discounting | N/A | | N/A |
| Different discount rates explored | N/A | | N/A |
| Uncertainty | X |  | Scenario analyses on time horizon, alternative sources for health utility values, cure point, costs, parametric functions for extrapolation, source for comparator. |
| Alternative payment models explored | N/A | | A Patient Access Scheme (PAS) was proposed by the submitting company and assessed by the Patient Access Scheme Assessment Group (PASAG) as acceptable for implementation in NHS Scotland. |

**Table 14 Checklist for assessing gene therapies. Drug: tisagenlecleucel-T (Kymriah) for treating relapsed or refractory diffuse large b-cell lymphoma. Setting: Italy (AIFA)**

| Item | Yes | No | Notes |
| --- | --- | --- | --- |
| Surrogate endpoint used | X |  | **Validation given?** N/A  Median OS data available but OS is not the primary endpoint. |
| Rare disease | N/A | | **Prevalence**: N/A  Only incidence data available. |
| Serious condition | X |  | The authority recognized high unmet need, lack of alternatives (and high toxicity profile of the few alternatives available). |
| Single-arm trial | X |  | **Matched historical cohort used?** Yes |
| Pediatric population |  | X | **Age range**: N/A |
| Reporting of adverse consequences and risks | X |  | N/A |
| Size of clinical trial | **Number of patients**: N/A | | N/A |
| Length of clinical trial | **Duration in months**: 13.9 | | Median follow-up at most updated cutoff at the time of the analysis. Maximum follow-up: 39 months. |
| Extrapolation to long-term outcomes | **Duration in months**: Lifetime horizon | | Parametric extrapolation. |
|  | **Yes** | **No** | **Quantification** |
| Severe disease | X |  | 26%-40.3% objective response rate and 6.3 overall survival with salvage chemotherapy. |
| Value to caregivers | N/A | | N/A |
| Insurance value | N/A | | N/A |
| Scientific spillovers | N/A | | N/A |
| Lack of alternatives | X |  | The authority recognized high unmet need, lack of alternatives (and high toxicity profile of the few alternatives available). |
| Substantial improvement in life expectancy | X |  | 51.6% objective response rate, 32.3% complete response. |
|  | **Yes** | **No** | **Notes** |
| Discounting | X |  | 3.5% on costs and outcomes per year. |
| Different discount rates explored |  | X | N/A |
| Uncertainty | X |  | Univariate, probabilistic, and scenario analysis. The authority requested additional scenario analyses with a different set of costs and different OS extrapolations. |
| Alternative payment models explored | N/A | | N/A |

**Table 15 Checklist for assessing gene therapies. Drug: tisagenlecleucel-T (Kymriah) for treating relapsed or refractory diffuse large b-cell lymphoma. Setting: France (HAS)**

| Item | Yes | No | Notes |
| --- | --- | --- | --- |
| Surrogate endpoint used | X |  | **Validation given?** N/A  Primary endpoint is complete response (OS data are also available but as part of secondary endpoints) |
| Rare disease | X |  | **Prevalence**: N/A  Orphan drug designation |
| Serious condition | X |  | High unmet need recognized, limited survival in case of relapsed / refractory disease |
| Single-arm trial | X |  | **Matched historical cohort used?** MAIC with SCHOLAR-1, PIX301, and Eyre 2016 studies. The HTA body expressed concerns about the effect size because of lack of direct comparison (the concerns remained also in the 2021 re-evaluation) |
| Pediatric population |  | X | **Age range**: adult patients |
| Reporting of adverse consequences and risks | X |  | The HTA body expressed concerns about short-term toxicity and lack of data about long-term adverse consequences (the concerns remained also in the 2021 re-evaluation) |
| Size of clinical trial | **Number of patients**: 115 actually treated, 165 ITT population;  167 ITT in 2020 | | N/A |
| Length of clinical trial | **Duration in months**: 40.3 (median follow-up for response rate in 2021) | | The HTA body expressed concerns about long-term effectiveness and the possibility of retreatment |
| Extrapolation to long-term outcomes | **Duration in months**: 120 (10 years) | | Independent parametric curves for OS extrapolation |
|  | **Yes** | **No** | **Quantification** |
| Severe disease | X |  | No explicit quantification, limited survival in case of relapsed / refractory disease |
| Value to caregivers | N/A | | N/A |
| Insurance value | N/A | | N/A |
| Scientific spillovers | N/A | | N/A |
| Lack of alternatives | X |  | The HTA body claimed that “very few alternatives exist” |
| Substantial improvement in life expectancy |  | X | The HTA body claimed that the treatment provides a minor improvement in the healthcare service provision based on complete remission and OS data (24% of patients in complete response after 40.3 months of median follow-up, 8.2 months median OS, 27% probability of being alive at 40.3 months |
|  | **Yes** | **No** | **Notes** |
| Discounting | X |  | 4% for both outcomes and costs |
| Different discount rates explored | X |  | In DSA |
| Uncertainty | X |  | Scenario analysis, DSA, PSA |
| Alternative payment models explored | N/A | | N/A |

**Table 16 Checklist for assessing gene therapies. Drug: tisagenlecleucel-T (Kymriah) for treating relapsed or refractory diffuse large b-cell lymphoma. Setting: Germany (G-BA)**

| Item | Yes | No | Notes |
| --- | --- | --- | --- |
| Surrogate endpoint used |  | X | **Validation given?** N/A |
| Rare disease | X |  | **Prevalence**: 450 – 720 patients  Orphan drug designation |
| Serious condition | N/A | | N/A |
| Single-arm trial | X |  | **Matched historical cohort used?** The manufacturer submitted various comparative data. The indirect comparison carried out was considered to be not sufficiently valid due to the lack of information on relevant confounders and thus insufficient adjustment. Further uncertainties exist in that 20% of the participants in the JULIET study do not fulfil the inclusion criteria of the SCHOLAR-1 study. In this respect, a sufficient adjustment is also not possible. An adequate comparison is therefore not available. |
| Pediatric population |  | X | **Age range**: adult patients |
| Reporting of adverse consequences and risks | X |  | Reporting is standard. However, the data are not sufficiently informative here, as they are selectively collected and not comparative. |
| Size of clinical trial | **Number of patients**: 167 | | N/A |
| Length of clinical trial | **Duration in months**: 32.6 | | Median observation time between inclusion and last study visit. The median time between the screening phase and the infusion of tisagenlecleucel was 115 days. This is significantly longer than the time period in clinical practice. |
| Extrapolation to long-term outcomes | N/A | | N/A |
|  | **Yes** | **No** | **Quantification** |
| Severe disease | N/A | | N/A |
| Value to caregivers | N/A | | N/A |
| Insurance value | N/A | | N/A |
| Scientific spillovers | N/A | | N/A |
| Lack of alternatives | N/A | | N/A |
| Substantial improvement in life expectancy | N/A | | The effect estimator is not in a magnitude in which one can derive an actual effect, taking into account the uncertainties. Therefore, the available data cannot be used to derive the magnitude of the added benefit. |
|  | **Yes** | **No** | **Notes** |
| Discounting | N/A | | N/A |
| Different discount rates explored | N/A | | N/A |
| Uncertainty | X |  | Hint |
| Alternative payment models explored | N/A | | Comprehensive quality assurance measures (prerequisites for the provision of therapy) have been specified. |

**Table 17 Checklist for assessing gene therapies. Drug: tisagenlecleucel (Kymriah) for treating relapsed or refractory b-cell acute lymphoblastic leukaemia in people aged up to 25 years. Setting: England (NICE)**

| Item | Yes | No | Notes |
| --- | --- | --- | --- |
| Surrogate endpoint used | X |  | **Validation given?** N/A  OS data available; however, OS was not the primary endpoint. The authority expressed concerns on maturity. |
| Rare disease | X |  | **Prevalence**: N/A  The manufacturer reported only incidence data. |
| Serious condition | X |  | Manufacturer, patient organizations, and expert opinion underlined the existence of unmet need. The authority did not dispute the claim. |
| Single-arm trial | X |  | **Matched historical cohort used?** Matched-adjusted indirect treatment comparison used. |
| Pediatric population | X |  | **Age range**: up to 25 years (pediatric and young adult population) |
| Reporting of adverse consequences and risks | X |  | The authority expressed concerns on the uncertainty surrounding adverse events and requested further scenario analysis in the cost-effectiveness model. |
| Size of clinical trial | **Number of patients**: 58  (ENSIGN trial) | | Size available only for one trial; the manufacturer requested data on the complete sample size to remain confidential. |
| Length of clinical trial | **Duration in months**: N/A | | The manufacturer requested data to remain confidential. |
| Extrapolation to long-term outcomes | **Duration in months**: 1,056 (88 years) | | At the time of submission, all 3 trials were still ongoing, the manufacturer requested data on median follow-up to remain confidential. The manufacturer used mixture cure and parametric models for base case. The authority expressed concerns on extrapolation methods for OS and requested additional scenario analyses. |
|  | **Yes** | **No** | **Quantification** |
| Severe disease | X |  | Life expectancy for untreated patients is < 24 months (median OS ranges from 3.9 to 7.5 months; median OS for treated patients is > 7.5 (the manufacturer did not disclose additional data on median OS). |
| Value to caregivers | X |  | No explicit quantification, only anectodical evidence from patient association and expert opinion. The authority did not dispute the claim. |
| Insurance value | N/A | | N/A |
| Scientific spillovers | X |  | No explicit quantification, only a claim based on expert opinion. The authority did not dispute the claim. |
| Lack of alternatives | X |  | No explicit quantification, only anectodical evidence from patient association and expert opinion. The authority did not dispute the claim. |
| Substantial improvement in life expectancy | X |  | The manufacturer claimed the curative nature of the treatment; ELIANA trial showed 81% complete response rate, 50% of the patients in disease-free survival at 12 months, median remission duration not reached. The authority expressed concerns on the curative nature of the treatment. |
|  | **Yes** | **No** | **Notes** |
| Discounting | X |  | 3.50% per annum discount rate for both costs and benefits. |
| Different discount rates explored | X |  | In scenario analyses. |
| Uncertainty | X |  | PSA, DSA, scenario analyses on mixture cure approach, standard parametric survival model, efficacy inputs, utility values, costs, time horizon and discounting factors. The authority requested additional scenario analyses on OS extrapolation, costs associated with non-infused patients, source of data to estimate efficacy of comparators, durations and costs of adverse events, post-transplant quality of life, health state utilities. |
| Alternative payment models explored | X |  | Managed entry agreement with evidence generation requirements. No explicit information on payment scheme. |

**Table 18** **Checklist for assessing gene therapies. Drug: tisagenlecleucel (Kymriah) for treating relapsed or refractory b-cell acute lymphoblastic leukaemia in people aged up to 25 years. Setting: United States (ICER)**

| Item | Yes | No | Notes |
| --- | --- | --- | --- |
| Surrogate endpoint used | X |  | **Validation given?** N/A  Overall remission rate as a surrogate for four-year event-free survival (preferred endpoint). The authority claimed that overall remission rate represents an optimistic presentation of the results that violates the intention to treat principle. |
| Rare disease | N/A | | **Prevalence**: N/A  over 3,000 new cases of ALL diagnosed in children and adolescents (ages 0-19) each year in the United States. |
| Serious condition | X |  | Fewer than one in three of these patients survive five years. |
| Single-arm trial | X |  | **Matched historical cohort used?** Naïve indirect comparisons. The authority acknowledges possible presence of selection bias; the studies are considered to be of lower quality because they lack comparators. |
| Pediatric population | X |  | **Age range**: 0-25 years (median age implemented in the economic model: 11.5 years, source: study B2202) |
| Reporting of adverse consequences and risks | X |  | N/A |
| Size of clinical trial | **Number of patients**: B2101J n = 55; B2205J n = 29; B2202/ELIANA n = 75 | | The authority claimed that small sample sizes add to the uncertainty for estimates of clinical efficacy. |
| Length of clinical trial | **Duration in months**: 8.7 months median follow-up in ELIANA trial | | The authority claimed that short median follow-up adds to the uncertainty for estimates of clinical efficacy. |
| Extrapolation to long-term outcomes | **Duration in months**: N/A | | The decision analytic model included a short-term decision tree and a long-term semi-Markov partitioned-survival model. Parametric extrapolation was used for PFS and OS. |
|  | **Yes** | **No** | **Quantification** |
| Severe disease | X |  | Literature data on impact on length and quality of life; high lifetime burden of illness. |
| Value to caregivers | N/A | | N/A |
| Insurance value | N/A | | N/A |
| Scientific spillovers | N/A | | N/A |
| Lack of alternatives | X |  | Relevance of the treatment for patients who failed other available treatments; this aspect was emphasized by patient organizations |
| Substantial improvement in life expectancy | X |  | Overall remission rate from 69% to 95%; 7.91 incremental life-years, 7.18 incremental QALYs when compared to clofarabine in the economic model. |
|  | **Yes** | **No** | **Notes** |
| Discounting | X |  | Costs and outcomes were discounted at 3% per year. |
| Different discount rates explored |  | X | N/A |
| Uncertainty | X |  | DSA, PSA, scenario analysis on different perspective, time horizons, parametric specification (introducing knots), absence of active comparator, outcomes-based reimbursement strategy, comparative static exercises on how acquisition cost should vary to achieve a set of given thresholds. The authority underlined the persistence of major uncertainty about the magnitude of the net health benefit compared to other therapies because there are no comparative trials and the existing single-arm trials are small with relatively short follow-up. |
| Alternative payment models explored | X |  | Value-based pricing suggested by the authority. At the time of the analysis, no public-policy coverages were available; private coverage policies required practitioners to generate evidence (e.g., failure of two tyrosine kinase inhibitors). |

**Table 19 Checklist for assessing gene therapies. Drug: tisagenlecleucel (Kymriah) for treating relapsed or refractory b-cell acute lymphoblastic leukaemia in people aged up to 25 years. Setting: Italy (AIFA)**

| Item | Yes | No | Notes |
| --- | --- | --- | --- |
| Surrogate endpoint used | X |  | **Validation given?** N/A  Median OS data available but OS is not the primary endpoint. |
| Rare disease | N/A | | **Prevalence**: N/A  Only incidence data available. |
| Serious condition | X |  | The authority recognized high unmet need due to poor outcomes with alternative treatments (chemotherapy). |
| Single-arm trial | X |  | **Matched historical cohort used?** Yes |
| Pediatric population | X |  | **Age range**: up to 25 years |
| Reporting of adverse consequences and risks | X |  | N/A |
| Size of clinical trial | **Number of patients**: N/A | | N/A |
| Length of clinical trial | **Duration in months**: 54.8 | | Maximum follow-up duration. No data available on median follow-up duration. |
| Extrapolation to long-term outcomes | **Duration in months**: Lifetime horizon | | Parametric extrapolation. |
|  | **Yes** | **No** | **Quantification** |
| Severe disease | X |  | Median OS is 7.7 months with standard chemotherapy. |
| Value to caregivers | N/A | | N/A |
| Insurance value | N/A | | N/A |
| Scientific spillovers | N/A | | N/A |
| Lack of alternatives | X |  | The authority recognized high unmet need, lack of alternatives (poor outcomes with the few alternatives available). |
| Substantial improvement in life expectancy | X |  | 81.3% complete remission rate, 66.2% of patients alive after 30 months. |
|  | **Yes** | **No** | **Notes** |
| Discounting | X |  | 3.5% on costs and outcomes per year. |
| Different discount rates explored |  | X | N/A |
| Uncertainty | X |  | Univariate, probabilistic, and scenario analysis. The authority requested additional scenario analyses with a different set of costs and different OS extrapolations. |
| Alternative payment models explored | N/A | | N/A |

**Table 20 Checklist for assessing gene therapies. Drug: tisagenlecleucel (Kymriah) for treating relapsed or refractory b-cell acute lymphoblastic leukaemia in people aged up to 25 years. Setting: France (HAS)**

| Item | Yes | | No | Notes |
| --- | --- | --- | --- | --- |
| Surrogate endpoint used | X | |  | **Validation given?** N/A  2018 analysis is based on complete response data; however, 2021 re-evaluation includes OS data |
| Rare disease | X | |  | **Prevalence**: N/A  810 new cases in France per year |
| Serious condition | X | |  | Complete recovery is rare (0-15%), overall survival is less than 6 months. The HTA body recognized high unmet need |
| Single-arm trial | X | |  | **Matched historical cohort used?** MAIC  The HTA body expressed concerns about the MAIC results |
| Pediatric population | X | |  | **Age range**: 3-21 years |
| Reporting of adverse consequences and risks | X | |  | The authority expressed concerns about the short-term toxicity profile and the absence of data on long-term possible adverse consequences, also after the 2021 update |
| Size of clinical trial | **Number of patients**: ELIANA: 97 included (79 treated)  ENSIGN: 73 included (58 treated). In 2021 75 included (64 treated) | | | N/A |
| Length of clinical trial | **Duration in months**: ELIANA: 8.4 (mean follow-up)  ENSIGN: 6  In 2021:  ELIANA: 30.2 (median follow-up for OS)  ENSIGN: 15.1 (median follow-up for OS) | | | The HTA body expressed concerns about long-term effectiveness even after the 2021 update |
| Extrapolation to long-term outcomes | **Duration in months**:  120 (10 years) | | | The HTA body expressed concerns about the selection method for parametric extrapolation (data used for external validation are based on a small sample size [n=2] and BIC and AIC criteria results were not presented) |
|  | **Yes** | | **No** | **Quantification** |
| Severe disease | X | |  | Complete recovery is rare (0-15%), overall survival is less than 6 months. The HTA body defined the condition as “severe” |
| Value to caregivers | N/A | | | N/A |
| Insurance value | N/A | | | N/A |
| Scientific spillovers | N/A | | | N/A |
| Lack of alternatives |  | | X | Salvage chemotherapy |
| Substantial improvement in life expectancy | X | |  | Superiority to salvage chemotherapy based on HRs from MAIC |
|  | **Yes** | | **No** | **Notes** |
| Discounting | X |  | | 4% applied to costs and outcomes |
| Different discount rates explored | X |  | | In DSA |
| Uncertainty | X | |  | DSA, PSA, scenario analysis |
| Alternative payment models explored | N/A | | | N/A |

**Table 21 Checklist for assessing gene therapies. Drug: tisagenlecleucel-T (Kymriah) for treating relapsed or refractory b-cell acute lymphoblastic leukaemia in people aged up to 25 years. Setting: Germany (G-BA)**

| Item | Yes | No | Notes |
| --- | --- | --- | --- |
| Surrogate endpoint used |  | X | **Validation given?** N/A |
| Rare disease | X |  | **Prevalence**: 50 – 65 patients  Orphan drug designation |
| Serious condition | N/A | | N/A |
| Single-arm trial | X |  | **Matched historical cohort used?** The manufacturer presented a MAIC as well as an indirect comparison. As there are uncertainties regarding the adjustment and the effect estimate is not at a level where one can derive an actual effect taking into account the uncertainties, no statement on the extent of the added benefit can be made on the basis of the available results. An adequate comparison is therefore not available. |
| Pediatric population |  | X | **Age range**: up to 25 years |
| Reporting of adverse consequences and risks | X |  | Reporting is standard. |
| Size of clinical trial | **Number of patients**: ELIANA: 97  ENSIGN: 75 | | N/A |
| Length of clinical trial | **Duration in months**: ELIANA: 24.9  ENSIGN: 13.6 | | Median observation time for OS |
| Extrapolation to long-term outcomes | N/A | | N/A |
|  | **Yes** | **No** | **Quantification** |
| Severe disease | N/A | | N/A |
| Value to caregivers | N/A | | N/A |
| Insurance value | N/A | | N/A |
| Scientific spillovers | N/A | | N/A |
| Lack of alternatives | N/A | | N/A |
| Substantial improvement in life expectancy | N/A | | As no comparative data are available, no statement on the extent of the added benefit can be made on the basis of the results. |
|  | **Yes** | **No** | **Notes** |
| Discounting | N/A | | N/A |
| Different discount rates explored | N/A | | N/A |
| Uncertainty | X |  | Hint |
| Alternative payment models explored | N/A | | Comprehensive quality assurance measures (prerequisites for the provision of therapy) have been specified. |

**Table 22 Checklist for assessing gene therapies. Drug: axicabtagene ciloleucel (Yescarta) for treating diffuse large b-cell lymphoma and primary mediastinal b-cell lymphoma after 2 or more systemic therapies. Setting: England (NICE)**

| Item | Yes | No | Notes |
| --- | --- | --- | --- |
| Surrogate endpoint used | X |  | **Validation given?** N/A  OS data available from ZUMA-1 trial (Phase I/II); however, it was not the primary endpoint. The authority expressed concerns on maturity. |
| Rare disease | N/A | | **Prevalence**: N/A  The manufacturer reported data on incidence only. |
| Serious condition | X |  | High unmet need in patients with refractory disease claimed by the manufacturer, patient organizations, and expert opinion. The authority did not dispute the claim. |
| Single-arm trial | X |  | **Matched historical cohort used?** Indirect and mixed treatment comparison performed on US registry data. The authority expressed concerns on possible bias arising from lack of data to control for a set of key variables. |
| Pediatric population |  | X | **Age range**: “adults”, median age in ZUMA-1 trial is 58 years. |
| Reporting of adverse consequences and risks | X |  | The authority expressed concerns on long-term adverse events management costs. |
| Size of clinical trial | **Number of patients**: 108 | |  |
| Length of clinical trial | **Duration in months**: 15.4 | | 15.4 months follow-up at the time of the submission. The authority expressed concerns on the immaturity of OS and PFS. |
| Extrapolation to long-term outcomes | **Duration in months**: 528 (44 years) | | The manufacturer implemented mixture cure models to extrapolate long-term efficacy. The authority expressed concerns on how the immaturity of OS data could affect the estimate of the cure fraction, base-case model was considered overly optimistic. |
|  | **Yes** | **No** | **Quantification** |
| Severe disease | X |  | Median OS without treatment ranges from 3.3 to 6.3 months and this is part of the argument used by the manufacturer to claim that NICE end-of-life criterion is met. |
| Value to caregivers | N/A | | N/A |
| Insurance value | N/A | | N/A |
| Scientific spillovers | N/A | | N/A |
| Lack of alternatives | X |  | No explicit quantification, only evidence from patient association and expert opinion. The authority did not dispute the claim. |
| Substantial improvement in life expectancy | X |  | 40% complete response, 52% overall survival at 18 months compared to 20% survival at 2 years in absence of treatment (expert opinion), curative intent. The authority expressed concerns on the assumption of cure at two years. |
|  | **Yes** | **No** | **Notes** |
| Discounting | X |  | 3.50% per annum discount rate for both costs and benefits. |
| Different discount rates explored | X |  | In scenario analyses. |
| Uncertainty | X |  | PSA, DSA, scenario analyses on different way historical cohort data were adjusted for baseline characteristics with respect to the pivotal clinical trial, time horizon, discounting factor, model type, parametric distributions for mixture cure model, best supportive care PFS, health state utilities. The authority requested additional scenario analyses on post-treatment stem cells transplant, long-term adverse events management costs, broader infrastructure and training requirements, and health state utilities. |
| Alternative payment models explored | X |  | Managed entry agreement with evidence generation requirements. No explicit information on payment scheme. |

**Table 23 Checklist for assessing gene therapies. Drug: axicabtagene ciloleucel (Yescarta) for treating diffuse large b-cell lymphoma in adults ineligible for autologous stem cells transplant. Setting: United States (ICER)**

| Item | Yes | No | Notes |
| --- | --- | --- | --- |
| Surrogate endpoint used | X |  | **Validation given?** N/A  Intermediate outcomes (i.e., complete remission, event-free survival, and objective response) were used to infer the key measure of benefit in the model (i.e., overall survival) |
| Rare disease | N/A | | **Prevalence**: N/A  25% of new diagnosed cases of non-Hodgkin lymphoma per year in the US |
| Serious condition | X |  | Five-year survival is approximately 50-70% |
| Single-arm trial | X |  | **Matched historical cohort used?** Yes, using observational data on salvage chemotherapy. The authority considered ZUMA-1 (pivotal study) to be of lower quality because it lacks comparators. |
| Pediatric population |  | X | **Age range**: 18 years and older |
| Reporting of adverse consequences and risks | X |  | N/A |
| Size of clinical trial | **Number of patients**: NCT00924326 n = 22, ZUMA-1 n = 101 | | The authority claimed that small sample sizes add to the uncertainty for estimates of clinical efficacy. |
| Length of clinical trial | **Duration in months**: Median follow-up of 15.4 months in ZUMA-1 clinical study | | The authority claimed that short median follow-up adds to the uncertainty for estimates of clinical efficacy. |
| Extrapolation to long-term outcomes | **Duration in months**: N/A | | The decision analytic model included a short-term decision tree and a long-term semi-Markov partitioned-survival model. Parametric extrapolation was used for PFS and OS. |
|  | **Yes** | **No** | **Quantification** |
| Severe disease | X |  | Literature data on impact on length and quality of life; high lifetime burden of illness. |
| Value to caregivers | N/A | | N/A |
| Insurance value | N/A | | N/A |
| Scientific spillovers | N/A | | N/A |
| Lack of alternatives | X |  | Relevance of the treatment for patients who failed other available treatments; this aspect was emphasized by patient organizations |
| Substantial improvement in life expectancy | X |  | Overall response rate: 82%; complete response rate: 54%; Complete remission rate 47%; 4.12 incremental life years, 3.40 incremental QALYs when compared to chemotherapy. |
|  | **Yes** | **No** | **Notes** |
| Discounting | X |  | Costs and outcomes were discounted at 3% per year. |
| Different discount rates explored |  | X | N/A |
| Uncertainty | X |  | PSA; scenario analysis on different perspective, time horizons, parametric specification (introducing knots), absence of active comparator, outcomes-based reimbursement strategy, comparative static exercises on how acquisition cost should vary to achieve a set of given thresholds. The authority underlined the persistence of major uncertainty about the magnitude of the net health benefit compared to other therapies because there are no comparative trials and the existing single-arm trials are small with relatively short follow-up. |
| Alternative payment models explored | X |  | Value-based pricing suggested by the authority. At the time of the analysis, no public-policy coverages were available; private coverage policies required practitioners to generate evidence (e.g., failure of two tyrosine kinase inhibitors). |

**Table 24 Checklist for assessing gene therapies. Drug: axicabtagene ciloleucel (Yescarta) for treating diffuse large b-cell lymphoma and primary mediastinal b-cell lymphoma after 2 or more systemic therapies. Setting: Scotland (SMC)**

| Item | Yes | No | Notes |
| --- | --- | --- | --- |
| Surrogate endpoint used | X |  | **Validation given?** N/A  Primary endpoint considered was objective response rate, median overall survival was not reached. |
| Rare disease | X |  | **Prevalence**: N/A  The treatment met the ultra-orphan criteria for the indication. |
| Serious condition | X |  | The authority recognized the condition as an aggressive form of non-Hodgkin lymphoma which entails poor outcomes for relapsed / refractory patients. The authority recognized high unmet need. |
| Single-arm trial | X |  | **Matched historical cohort used?** Unanchored indirect comparison with patient level data from an international, multi-cohort retrospective study of patients with refractory DLBCL (including PMBCL and TFL), to compare axicabtagene ciloleucel with salvage chemotherapy. The authority expressed concerns on the weaknesses of the indirect comparison method. |
| Pediatric population |  | X | **Age range**: N/A |
| Reporting of adverse consequences and risks | X |  | N/A |
| Size of clinical trial | **Number of patients**: 101 | | N/A |
| Length of clinical trial | **Duration in months**: 27.1 | | Median follow-up in ZUMA-1 (pivotal clinical study). |
| Extrapolation to long-term outcomes | **Duration in months**: N/A | | Lifetime horizon. |
|  | **Yes** | **No** | **Quantification** |
| Severe disease | X |  | Overall survival of 6.3 months and a two-year survival rate of 20% if treated with standard of care. SMC end-of-life criterion is met. |
| Value to caregivers | X |  | According to patient organizations, the single-infusion feature is an advantage for caregivers and the treatment benefits could reduce the caring duties of family and carers. |
| Insurance value | N/A | | N/A |
| Scientific spillovers | N/A | | N/A |
| Lack of alternatives | X |  | Expert opinion underlined very limited treatment options and poor outcomes in adult patients with relapsed or refractory DLBCL. |
| Substantial improvement in life expectancy | X |  | The authority deemed the treatment “life-extending” |
|  | **Yes** | **No** | **Notes** |
| Discounting | N/A | | N/A |
| Different discount rates explored | N/A | | N/A |
| Uncertainty | X |  | Different parametrizations for OS and PFS, different approaches to estimate utility, different time horizons, different costs, and proportion of patients experiencing curative effect. |
| Alternative payment models explored | N/A | | A Patient Access Scheme (PAS) was submitted by the company and assessed by the Patient Access Scheme Assessment Group (PASAG) as acceptable for implementation in NHS Scotland |

**Table 25 Checklist for assessing gene therapies. Drug: axicabtagene ciloleucel (Yescarta) for treating diffuse large b-cell lymphoma and primary mediastinal b-cell lymphoma after 2 or more systemic therapies. Setting: Italy (AIFA)**

| Item | Yes | No | Notes |
| --- | --- | --- | --- |
| Surrogate endpoint used | X |  | **Validation given?** N/A  OS not reached, evaluation was based on response rate and PFS |
| Rare disease | X |  | **Prevalence**: N/A, only incidence available.  The drug was negotiated according to the “orphan drug for rare disease” procedure |
| Serious condition | X |  | High unmet need recognized |
| Single-arm trial | X |  | **Matched historical cohort used?** Yes, SCHOLAR-1 study |
| Pediatric population |  | X | **Age range**: adult population, incidence increases with age |
| Reporting of adverse consequences and risks | X |  | The HTA body expressed concerns about the fact that adverse events were not included in the economic model, except for cytokine release syndrome for Yescarta arm |
| Size of clinical trial | **Number of patients**: 108 | |  |
| Length of clinical trial | **Duration in months**: 27.1 | | Median follow-up |
| Extrapolation to long-term outcomes | **Duration in months**: 504 (42 years, lifetime perspective) | | The HTA body requested to modify the assumptions on survival curves extrapolation |
|  | **Yes** | **No** | **Quantification** |
| Severe disease | N/A | | N/A |
| Value to caregivers | N/A | |  |
| Insurance value | N/A | |  |
| Scientific spillovers | N/A | |  |
| Lack of alternatives | X |  | N/A |
| Substantial improvement in life expectancy | X |  | 83% overall response rate, 58% complete response rate, +4.16 incremental life years gained, +3.35 incremental QALYs gained compared to best supportive care |
|  | **Yes** | **No** | **Notes** |
| Discounting | X |  | 3.5% for costs and benefits |
| Different discount rates explored | X |  | The HTA body explicitly requested to explore different discount rates |
| Uncertainty | X |  | Cost-effectiveness Acceptability Curve plotted |
| Alternative payment models explored | X |  | Payment at result scheme (at 180, 270, and 365 days) on top of confidential discount |

**Table 26 Checklist for assessing gene therapies. Drug: axicabtagene ciloleucel (Yescarta) for treating diffuse large b-cell lymphoma and primary mediastinal b-cell lymphoma after 2 or more systemic therapies. Setting: France (HAS)**

| Item | Yes | No | Notes |
| --- | --- | --- | --- |
| Surrogate endpoint used | X |  | **Validation given?** N/A  The analysis is based primarily on complete response data (OS data are also used as secondary endpoint) |
| Rare disease | X |  | **Prevalence**: N/A  The treatment is evaluated under the orphan drug designation |
| Serious condition | X |  | High unmet need recognized, limited survival in relapsed / refractory setting |
| Single-arm trial | X |  | **Matched historical cohort used?** Yes (indirect comparison with SCHOLAR-1 observational study data)  The HTA body expressed concerns about lack of direct comparison, method used for indirect comparison (propensity score matching), and quality of data. These concerns persist in 2021 re-evaluation |
| Pediatric population |  | X | **Age range**: 18 years or older |
| Reporting of adverse consequences and risks | X |  | The HTA body expressed concerns about short-term toxicity and lack of long-term data on safety (these concerns persist in 2021 re-evaluation) |
| Size of clinical trial | **Number of patients**: 111 ITT, 101 received administration in 2017 | | N/A |
| Length of clinical trial | **Duration in months**: 8.7 (median follow-up in 2017)  27.1 (median follow-up in 2020) | | The HTA body expressed concerns about long-term effectiveness (these concerns persist in 2021 re-evaluation) |
| Extrapolation to long-term outcomes | **Duration in months**: 408 (32 years, lifetime horizon) | | The HTA body expressed concerns on OS and PFS extrapolation (in particular the hypothesis that a portion of patients is considered cured). The HTA body requested a base-case analysis with a 20-year time horizon |
|  | **Yes** | **No** | **Quantification** |
| Severe disease | X |  | No explicit quantification, limited survival in relapsed / refractory setting |
| Value to caregivers | N/A | | N/A |
| Insurance value | N/A | | N/A |
| Scientific spillovers | N/A | | N/A |
| Lack of alternatives | X |  | The HTA body claimed the existence of “very few alternatives” |
| Substantial improvement in life expectancy | X |  | The HTA body claimed the treatment provides a moderate improvement in healthcare service delivery based on objective response rate (68% of patients in complete response in 2021) and OS (median 17.4 months in 2021 |
|  | **Yes** | **No** | **Notes** |
| Discounting | X |  | 4% for costs and outcomes |
| Different discount rates explored | X |  | In DSA |
| Uncertainty | X |  | Scenario analysis, DSA, PSA |
| Alternative payment models explored |  | | N/A |

**Table 27 Checklist for assessing gene therapies. Drug: axicabtagene ciloleucel (Yescarta) for treating diffuse large b-cell lymphoma and primary mediastinal b-cell lymphoma after 2 or more systemic therapies. Setting: Germany (G-BA)**

| Item | Yes | No | Notes |
| --- | --- | --- | --- |
| Surrogate endpoint used |  | X | **Validation given?** N/A |
| Rare disease | X |  | **Prevalence**: DLBCL: 440 – 700 patients; PMBCL: 5 - 9 patients  Orphan drug designation |
| Serious condition | N/A | | N/A |
| Single-arm trial | X |  | **Matched historical cohort used?** The indirect comparison was used for the assessment of the endpoint mortality. However, due to the indirect historical comparison and due to further relevant uncertainties, a valid quantification of the magnitude of the effect on OS was not possible. |
| Pediatric population | X |  | **Age range**: up to 25 years |
| Reporting of adverse consequences and risks | X |  | Reporting is standard. |
| Size of clinical trial | **Number of patients**: 111 | | N/A |
| Length of clinical trial | **Duration in months**: 23.5 | | Median time between infusion and death or last date the patient was still alive |
| Extrapolation to long-term outcomes | N/A | | N/A |
|  | **Yes** | **No** | **Quantification** |
| Severe disease | N/A | | N/A |
| Value to caregivers | N/A | | N/A |
| Insurance value | N/A | | N/A |
| Scientific spillovers | N/A | | N/A |
| Lack of alternatives | N/A | | N/A |
| Substantial improvement in life expectancy | N/A | | Not quantifiable added benefit. However, the G-BA states that against the background of the far advanced stage of the disease and treatment as well as the poor prognosis for the further course of the disease, a high value is placed on the comparative results for overall survival in the overall assessment. |
|  | **Yes** | **No** | **Notes** |
| Discounting | N/A | | N/A |
| Different discount rates explored | N/A | | N/A |
| Uncertainty | X |  | Hint |
| Alternative payment models explored | N/A | | Comprehensive quality assurance measures (prerequisites for the provision of therapy) have been specified. |

**Table 28 Checklist for assessing gene therapies. Drug: voretigene neparvovec (Luxturna) for treating inherited retinal dystrophies caused by rpe65 gene mutations. Setting: England (NICE)**

| Item | Yes | No | Notes |
| --- | --- | --- | --- |
| Surrogate endpoint used | X |  | **Validation given?** N/A  The manufacturer provided literature evidence of visual acuity as surrogate endpoint for excess mortality in individuals with visual impairment; however, the authority expressed concerns that it was based on findings of a study conducted in elderly patients without *RPE65*-mediated IRD. Notice that life expectancy was not the primary endpoint. |
| Rare disease | X |  | **Prevalence**: 12.3-28.8 per 100,000 people. |
| Serious condition | X |  | Large unmet need stated by manufacturer, patient organizations, and expert opinion. The authority did not dispute the claim. |
| Single-arm trial |  | X | **Matched historical cohort used?** No |
| Pediatric population | N/A | | **Age range**: N/A  The drug is licensed for both pediatric and adult population. However, mean age in the pivotal clinical trial was 15.1 years, reflecting early onset of the disease. |
| Reporting of adverse consequences and risks | X |  | The authority expressed concerns on the lack of data on need for cataract surgery. |
| Size of clinical trial | **Number of patients**: 29 | | The authority claimed that the small sample size of the clinical study, although reflective of the rare nature of the condition, was a key limitation. |
| Length of clinical trial | **Duration in months**: 48 (4 years) | | A four-year follow-up was available for some, though not all, outcomes in this submission. |
| Extrapolation to long-term outcomes | **Duration in months**: N/A | | The economic model featured a lifetime horizon (up to 100 years). |
|  | **Yes** | **No** | **Quantification** |
| Severe disease | X |  | No explicit quantification, only literature evidence of the negative impact of the disease on the quality of life of patients. The authority expressed concerns on the HRQoL data used in the economic analysis. |
| Value to caregivers | X |  | No explicit quantification, only literature evidence of the negative impact of the disease on the quality of life of patients, plus anecdotal evidence for patient organizations. The authority claimed that lack of HRQoL data, or PRO data for the carers of patients from the pivotal trial was an important omission. |
| Insurance value | N/A | | N/A |
| Scientific spillovers | X |  | No explicit quantification, both manufacturer and expert opinion claimed that the technology could lead the way for gene therapy treatments for other more complicated causes of genetic blindness. The authority did not dispute the claim. |
| Lack of alternatives | X |  | Manufacturer claim (consistent with arguments for unmet need). The authority did not dispute the claim. |
| Substantial improvement in life expectancy |  |  | Life expectancy is not the primary endpoint. |
|  | **Yes** | **No** | **Notes** |
| Discounting | X |  | 3.50% per annum discount rate for both costs and benefits. |
| Different discount rates explored | X |  | In scenario analyses. |
| Uncertainty | X |  | PSA, DSA, scenario analyses on perspective, discount rate, eye approach, health state definition, source of baseline characteristics, duration of treatment, waning effect, etc. The authority suggested further sensitivity analysis on expected long-term treatment effects, health-related quality of life values to be tested, and modelling of the natural history of the condition. |
| Alternative payment models explored |  | X | Further data collection arrangement as part of a managed access agreement (shorter than 15 years) was deemed of limited added value. |

**Table 29** **Checklist for assessing gene therapies. Drug: voretigene neparvovec (Luxturna) for treating inherited retinal dystrophies caused by rpe65 gene mutations. Setting: United States (ICER)**

| Item | Yes | No | Notes |
| --- | --- | --- | --- |
| Surrogate endpoint used | X |  | **Validation given?** N/A  Intermediate outcomes (i.e., structural/functional tests of the retina, mobility tests) were linked to key measures of clinical benefit (i.e., quality of life, daily functioning). The authority recognized that the primary endpoint in the pivotal trial (i.e., the multi-luminance mobility test) was novel at the time of the analysis and major uncertainty existed on how scores in the multi-luminance mobility test translated to improvements in daily functioning. |
| Rare disease | X |  | **Prevalence**: unknown, preliminary estimates suggested between 1,000 and 3,000 individuals in the US. It is categorized among the ultra-rare conditions. |
| Serious condition | X |  | The condition is significantly related to blindness in adulthood. |
| Single-arm trial |  | X | **Matched historical cohort used?** No  Phase III pivotal trial was structured in a treatment arm and a delayed intervention arm (i.e., to no treatment with the option for treatment after one year). |
| Pediatric population | N/A | | **Age range**: N/A  Evidence suggests early onset of the condition, average age of individuals enrolled in the pivotal trial was 15 years. However, age ranged up to 44 years and the authority expressed concerns on heterogeneity of efficacy depending on age, youngest individuals being favored and that the pivotal phase III trial underrepresented older patients if compared to the phase I trial. |
| Reporting of adverse consequences and risks | X |  | N/A |
| Size of clinical trial | **Number of patients**: 31 | | 21 in the treatment arm and 10 in the delayed treatment arm. |
| Length of clinical trial | **Duration in months**: 36 | | 48 months for a limited set of outcomes. |
| Extrapolation to long-term outcomes | **Duration in months**: N/A | | Two-state Markov model starting at 15 years and covering a lifetime horizon. Extrapolation performed using parametric curves (exponential). |
|  | **Yes** | **No** | **Quantification** |
| Severe disease | X |  | No explicit quantification, only literature evidence that the condition diminishes the quality of life significantly. |
| Value to caregivers | X |  | No explicit quantification, only speculation that improved independence reduces the burden on caregivers. |
| Insurance value | N/A | | N/A |
| Scientific spillovers | X |  | No explicit quantification, only speculation that the treatment is expected to change the treatment paradigm, fostering improvements in screening. |
| Lack of alternatives | X |  | Literature evidence on absence of therapies to alter vision loss in individuals with that condition. |
| Substantial improvement in life expectancy | N/A | | The authority recognized that the treatment significantly improved mobility; however, it is not clear how it translates to life expectancy. Model-based economic evaluation estimated between 8.1 and 10.6 incremental blindness-free years. |
|  | **Yes** | **No** | **Notes** |
| Discounting | X |  | 3% per annum for both costs and outcomes. |
| Different discount rates explored |  | X | N/A |
| Uncertainty | X |  | DSA, PSA, scenario analysis varying the age at baseline, perspective, different source for utility values; threshold analysis varying the price of the technology to meet a set of cost-effectiveness thresholds. The authority expressed concerns on uncertainty surrounding quality of life estimates due to paucity of data on the specific condition. |
| Alternative payment models explored |  | X | The authority recommended payers, manufacturer and public provider to cooperate to develop payment mechanisms to improve access to the treatment. |

**Table 30 Checklist for assessing gene therapies. Drug: voretigene neparvovec (Luxturna) for treating inherited retinal dystrophies caused by rpe65 gene mutations. Setting: Canada (CADTH)**

| Item | Yes | No | Notes |
| --- | --- | --- | --- |
| Surrogate endpoint used | X |  | **Validation given?** N/A  The primary outcome of the pivotal trial (i.e., bilateral multi-luminance mobility test performance) was associated to improved independence and quality of life. |
| Rare disease | X |  | **Prevalence**: N/A  The authority expressed concerns on uncertainty generated by the rarity of the condition and relatively small sample size of the pivotal clinical study. |
| Serious condition | X |  | High unmet need, the treatment is the first one approved in Canada that targets the underlying mechanism of the disease. |
| Single-arm trial |  | X | **Matched historical cohort used?** No  The authority expressed concerns on uncertainty generated by the lack of active comparator. |
| Pediatric population | N/A | | **Age range**: N/A  The population consists of both children and adults; however, the onset often takes place during childhood. |
| Reporting of adverse consequences and risks | X |  | The authority underlined that although the benefit-risk profile appears to be acceptable, some patients may experience serious adverse events related to the procedure. |
| Size of clinical trial | **Number of patients**: 31 | | The authority expressed concerns on uncertainty generated by the rarity of the condition and relatively small sample size of the pivotal clinical study. |
| Length of clinical trial | **Duration in months**: 12 | | The authority expressed concerns on uncertainty generated by the limited duration of the pivotal clinical study and the fact that the majority of the modelled benefits were accrued in time periods beyond when clinical data are available. |
| Extrapolation to long-term outcomes | **Duration in months**: N/A | | Parametric multistate models. The authority expressed concerns on uncertainty generated by the limited duration of the pivotal clinical study and the fact that the majority of the modelled benefits were accrued in time periods beyond when clinical data are available. |
|  | **Yes** | **No** | **Quantification** |
| Severe disease | X |  | The progressive nature of the condition which leads to permanent vision loss |
| Value to caregivers | N/A | | N/A |
| Insurance value | N/A | | N/A |
| Scientific spillovers | N/A | | N/A |
| Lack of alternatives | X |  | The treatment is the first one approved in Canada that targets the underlying mechanism of the disease. |
| Substantial improvement in life expectancy | N/A | | The treatment demonstrated statistically significant improvement in functional vision under low light and it would likely be considered meaningful to patients. |
|  | **Yes** | **No** | **Notes** |
| Discounting | N/A | | N/A |
| Different discount rates explored | N/A | | N/A |
| Uncertainty | X |  | No detail on how the manufacturer characterized uncertainty in the economic evaluation model submitted. The authority expressed concerns on estimates of the duration of benefits due to the limited clinical follow-up (40 years was considered to be overly optimistic). Utility weights were also considered a major source of uncertainty. |
| Alternative payment models explored | X |  | The authority suggested a price reduction of more than 74% to meet the standard cost-effectiveness threshold ($50,000 per QALY) and product listing agreements at the single jurisdiction level. |

**Table 31 Checklist for assessing gene therapies. Drug: voretigene neparvovec (Luxturna) for treating inherited retinal dystrophies caused by rpe65 gene mutations. Setting: Scotland (SMC)**

| Item | Yes | No | Notes |
| --- | --- | --- | --- |
| Surrogate endpoint used | X |  | **Validation given?** N/A  The primary endpoint was an endpoint created ad hoc (mean change from baseline to one year in bilateral multi-luminance mobility test (MLMT)); additionally, patients were requested to complete a visual function questionnaire. The authority expressed concerns on the fact that MLMT was not suitable for the economic analysis because it cannot be linked to costs; as a consequence, secondary and exploratory outcomes (visual acuity and visual functioning) were employed. Another concern was on how visual function questionnaire data could be linked to quality of life. |
| Rare disease | X |  | **Prevalence**: <1 in 50,000 of the population in Scotland  The treatment met SMC ultra-orphan criteria. |
| Serious condition | X |  | The authority recognized chronicity, severity and high unmet need for the condition. |
| Single-arm trial |  | X | **Matched historical cohort used?** No  Phase III pivotal trial was structured in a treatment arm and a delayed intervention arm (i.e., to no treatment with the option for treatment after one year). |
| Pediatric population | N/A | | **Age range**: adult and pediatric population.  The condition tends to be characterized by early onset; however, progression to complete blindness can occur from pre-school to the third decade of life. |
| Reporting of adverse consequences and risks | X |  | The authority expressed concerns on lack of data on long-term consequences. |
| Size of clinical trial | **Number of patients**: 31 | | N/A |
| Length of clinical trial | **Duration in months**: up to 48 | | Maximum follow-up duration was 4 years. The authority expressed concerns on how the limited duration of the follow-up could affect the estimates of treatment effect. The follow-up on the pivotal trial is expected to be 15 years. |
| Extrapolation to long-term outcomes | **Duration in months**: lifetime horizon | | The manufacturer submitted a parametric survival model. The authority expressed concerns on the assumption that treatment duration was 40 years. |
|  | **Yes** | **No** | **Quantification** |
| Severe disease | X |  | The authority recognized chronicity and severity of the condition, and its effect in impairing education, labor market, and socializing activities. |
| Value to caregivers | X |  | Patient organizations claimed that the treatment has the potential to improve independence, hence reducing the burden on caregivers. The authority did not dispute the claim. |
| Insurance value | N/A | | N/A |
| Scientific spillovers | N/A | | N/A |
| Lack of alternatives | X |  | The authority recognized the absence of medicines licensed for this condition. |
| Substantial improvement in life expectancy | X |  | The authority recognized a significant improvement in MLMT; it also acknowledged that it may be underestimated due to ceiling effect. |
|  | **Yes** | **No** | **Notes** |
| Discounting | N/A | | N/A |
| Different discount rates explored | N/A | | N/A |
| Uncertainty | X |  | The manufacturer implemented deterministic sensitivity analysis on a number of parameters in the economic model. The authority expressed concerns on the uncertainty generated by the health utility scores and transition probabilities utilized. |
| Alternative payment models explored | N/A | | A patient access scheme was submitted by the company and assessed by the Patient Access Scheme Assessment Group (PASAG) as acceptable for implementation in NHS Scotland. The authority expressed concerns on the financial impact of the high upfront costs related to the uncertainty on long-term benefits. |

**Table 32 Checklist for assessing gene therapies. Drug: voretigene neparvovec (Luxturna) for treating inherited retinal dystrophies caused by rpe65 gene mutations. Setting: France (HAS)**

| Item | Yes | No | Notes |
| --- | --- | --- | --- |
| Surrogate endpoint used | X |  | **Validation given?** The primary endpoint (variation on MLMT score) was deemed relevant according to the HTA body |
| Rare disease | X |  | **Prevalence**: 1.8/100,000 for Leber's hereditary optic neuropathy, 1/5,000 – 1/3,000 for pigmentary retinitis |
| Serious condition | X |  | High unmet need recognized |
| Single-arm trial |  | X | **Matched historical cohort used?** N/A  Comparative phase III study against absence of treatment. The HTA body expressed concerns about the fact that the study is open-label |
| Pediatric population | X |  | **Age range**: N/A  Onset tends to happen early in life |
| Reporting of adverse consequences and risks | X |  | The HTA body expressed concerns about the lack of long-term safety data |
| Size of clinical trial | **Number of patients**: 31 | |  |
| Length of clinical trial | **Duration in months**: At least 24 (2 patients had a 36 -month follow-up and 4 patients had a 48-month follow-up) | | The HTA body expressed concerns about the lack of long-term effectiveness data |
| Extrapolation to long-term outcomes | **Duration in months**: Lifetime (patients enter the model at 3 years and can live up to 100 years) | | The model is based on parametric extrapolation (lognormal).  The HTA body expressed concerns about the hypothesis on which the derivation of the transition probabilities for a specific state (HS5) is based upon. |
|  | **Yes** | **No** | **Quantification** |
| Severe disease | X |  | No explicit quantification, the HTA body recognized the severity of the disease and defined it as “disabling and leading to blindness” |
| Value to caregivers | N/A | | N/A |
| Insurance value | N/A | | N/A |
| Scientific spillovers | N/A | | N/A |
| Lack of alternatives | X |  | No existing alternative |
| Substantial improvement in life expectancy | N/A | | Improving life expectancy is not the objective of the treatment |
|  | **Yes** | **No** | **Notes** |
| Discounting | X |  | 4% |
| Different discount rates explored | X |  | 0%, 2.5%, 4% |
| Uncertainty | X |  | Scenario analysis, PSA, DSA |
| Alternative payment models explored | N/A | | The proposed reimbursement rate is 65%. It can be inferred that a confidential discount was applied but it is not explicitly stated |

**Table 33 Checklist for assessing gene therapies. Drug: voretigene neparvovec (Luxturna) for treating inherited retinal dystrophies caused by rpe65 gene mutations. Setting: Germany (G-BA)**

| Item | Yes | No | Notes |
| --- | --- | --- | --- |
| Surrogate endpoint used |  | X | **Validation given?** N/A |
| Rare disease | X |  | **Prevalence**: 100 - 530 patients  Orphan drug designation |
| Serious condition | N/A | | N/A |
| Single-arm trial |  | X | **Matched historical cohort used?** N/A |
| Pediatric population | X |  | **Age range**: pediatric and adult patients |
| Reporting of adverse consequences and risks | X |  | Reporting is standard. |
| Size of clinical trial | **Number of patients**: 31 | | N/A |
| Length of clinical trial | **Duration in months**: 12 | | Observation time for adverse events |
| Extrapolation to long-term outcomes | N/A | | N/A |
|  | **Yes** | **No** | **Quantification** |
| Severe disease | N/A | | N/A |
| Value to caregivers | N/A | | N/A |
| Insurance value | N/A | | N/A |
| Scientific spillovers | N/A | | N/A |
| Lack of alternatives | N/A | | N/A |
| Substantial improvement in life expectancy |  | X | No deaths |
|  | **Yes** | **No** | **Notes** |
| Discounting | N/A | | N/A |
| Different discount rates explored | N/A | | N/A |
| Uncertainty | X |  | Hint |
| Alternative payment models explored | N/A | | N/A |

**Table 34** **Checklist for assessing gene therapies. Drug: Strimvelis for treating severe combined immunodeficiency caused by adenosine deaminase deficiency. Setting: England (NICE)**

| Item | Yes | No | Notes |
| --- | --- | --- | --- |
| Surrogate endpoint used | X |  | **Validation given?** N/A  Overall survival (OS) is measured as endpoint in the pivotal clinical study; however, it was not the primary endpoint. The authority expressed concerns on potential overestimation of OS due to concomitant use of other therapies and preferred intervention-free survival as primary endpoint. |
| Rare disease | X |  | **Prevalence**: N/A  Incidence 1:200,000 to 1:1 million (3 new cases per year in England). |
| Serious condition | X |  | The authority recognized it as a life-shortening condition impacting all aspects of life of patients are caregivers. |
| Single-arm trial | X |  | **Matched historical cohort used?** Yes, hematopoietic stem cell transplant cohort. The authority expressed concerns on variable reporting and lack of comparable data for many outcomes. |
| Pediatric population | X |  | **Age range**: 0.5 – 6.1 years, median 1.7 in pivotal trial.  The authority expressed concerns that the age of people treated in the study is older than the expected age of people who are newly diagnosed. |
| Reporting of adverse consequences and risks | X |  | N/A |
| Size of clinical trial | **Number of patients**: 18 | | The authority expressed concerns on uncertainty based on the small sample size. |
| Length of clinical trial | **Duration in months**: N/A | | The authority expressed concerns on small number of deaths during the follow-up time, which impacted efficacy estimates. |
| Extrapolation to long-term outcomes | **Duration in months**: N/A | | Long-term survival extrapolated over a lifetime time horizon using a Markov modelling approach. The authority agreed on suitability of long-term extrapolation. |
|  | **Yes** | **No** | **Quantification** |
| Severe disease | X |  | No explicit quantification, only literature evidence on the impact of infections. |
| Value to caregivers | X |  | No explicit quantification, only literature estimates of quality of life of family members and anecdotical evidence from patient associations. The authority did not dispute the claim. |
| Insurance value | N/A | | N/A |
| Scientific spillovers | N/A | | N/A |
| Lack of alternatives | N/A | | N/A |
| Substantial improvement in life expectancy | X |  | Incremental QALYs in the economic model ranged from 11.7 to 13.6 . The manufacturer requested survival results from the pivotal trial to remain confidential. The authority did not dispute the claim of longer survival compared to transplant; however, the authority expressed concerns on uncertainty on the size of clinical benefits. |
|  | **Yes** | **No** | **Notes** |
| Discounting | X |  | Both 1.5% and 3.5% per annum for costs and outcomes. The authority accepted the manufacturer’s request to use 1.5% per annum for base case because the treatment requires a high upfront cost and grants expected lifetime benefits. |
| Different discount rates explored | X |  | See above. |
| Uncertainty | X |  | The manufacturer implemented sensitivity analysis on joint uncertainties in long-term utility scores and mean life-expectancy for survivors. The authority expressed concerns on potential underestimation of costs for the treatment (and overestimation of costs for the comparator), overestimation of overall survival. The authority proposed an alternative set of costs to be tested. |
| Alternative payment models explored |  | X | Due to low ICERs, budget impact, restricted indication, and data collection mechanisms already in place, managed entry agreements were deemed not necessary. |

**Table 35** **Checklist for assessing gene therapies. Drug: talimogene laherparepvec (T-VEC) (Imlygic) for treating unresectable metastatic melanoma. Setting: England (NICE)**

| Item | Yes | No | Notes |
| --- | --- | --- | --- |
| Surrogate endpoint used | X |  | **Validation given?** No  Durable response rate (DRR) is the primary endpoint of the pivotal clinical trial (OPTiM); overall survival is the key secondary endpoint. The authority expressed concerns that DRR is non-validated and potentially prone to bias |
| Rare disease | N/A | | **Prevalence**: N/A  13,348 new cases diagnosed in 2011 in UK |
| Serious condition | X |  | Low survival rates, negative impact on productivity, significant unmet need due to lack of durable response and toxicity of existing treatments were pointed out by manufacturer, experts and patient associations; the authority did not dispute the claims |
| Single-arm trial |  | X | **Matched historical cohort used?** No  Due to absence of head-to-head comparison with a set of relevant comparators, the manufacturer proposed indirect comparisons using Korn and two-step modified Korn models and using American Joint Committee on Cancer survival trends. The authority expressed concerns on the methods used for the indirect comparison and the lack of consideration of a relevant comparator (pembrolizumab) |
| Pediatric population |  | X | **Age range**: adults (age ≥ 18 years) |
| Reporting of adverse consequences and risks | X |  | The authority expressed concerns on lack of data supporting the long-term safety of the treatment |
| Size of clinical trial | **Number of patients**: 436 | | N/A |
| Length of clinical trial | **Duration in months**: 48 (4 years) | | Data cut of OS was conducted when all subjects had been followed for at least 3 years. The authority expressed concerns on the maturity of OS data used for the economic evaluation |
| Extrapolation to long-term outcomes | **Duration in months**: 360 (30 years) | | The manufacturer proposed parametric extrapolation. The authority expressed concerns on the maturity of OS data used for the economic evaluation |
|  | **Yes** | **No** | **Quantification** |
| Severe disease | X |  | Without treatment, patients are expected to survive between 3-9 months; however, the applicability of NICE end-of-life criteria was not discussed. Manufacturer and patient organizations emphasized the productivity loss (no quantification); the authority did not dispute the claim |
| Value to caregivers | N/A | | N/A |
| Insurance value | N/A | | N/A |
| Scientific spillovers | N/A | | N/A |
| Lack of alternatives | X |  | The treatment is first in class and it was recognized the toxicity of possible alternative treatments |
| Substantial improvement in life expectancy | X |  | 1.76 incremental LYG and 1.34 incremental QALYs compared to ipilimumab in base-case analysis |
|  | **Yes** | **No** | **Notes** |
| Discounting | X |  | Costs and outcomes were discounted at 3.5% per annum |
| Different discount rates explored | X |  | Different discounts explored in DSA |
| Uncertainty | X |  | PSA, DSA, scenario analyses varying time horizon, modelling approach for treatment and comparator, alternative sources for utility estimation, alternative parametric curve fit, resource use in terminal care, routine treatment for non-progressive treatment |
| Alternative payment models explored |  | X | Patient access scheme based on discount to list price applied at point of purchase or invoice |

**Table 36 Checklist for assessing gene therapies. Drug: talimogene laherparepvec (T-VEC) (Imlygic) for treating unresectable metastatic melanoma. Setting: Germany (G-BA)**

| Item | Yes | | No | Notes |
| --- | --- | --- | --- | --- |
| Surrogate endpoint used |  | | X | **Validation given?** N/A |
| Rare disease |  | | X | **Prevalence**: Non-pretreated patients with a BRAFV600-mutated tumour: 35 - 100 patients; non-pretreated patients with a BRAFV600 wild-type tumour: 40 - 120 patients; pre-treated patients: 300 - 450 patients |
| Serious condition | N/A | | | N/A |
| Single-arm trial |  | | X | **Matched historical cohort used?** N/A |
| Pediatric population |  | | X | **Age range**: pediatric and adult patients |
| Reporting of adverse consequences and risks |  | X | | No trial available that fulfilled PICOS-criteria |
| Size of clinical trial | **Number of patients**: N/A | | | No trial available that fulfilled PICOS-criteria |
| Length of clinical trial | **Duration in months**: N/A | | | No trial available that fulfilled PICOS-criteria |
| Extrapolation to long-term outcomes | N/A | | | N/A |
|  | **Yes** | | **No** | **Quantification** |
| Severe disease | N/A | | | N/A |
| Value to caregivers | N/A | | | N/A |
| Insurance value | N/A | | | N/A |
| Scientific spillovers | N/A | | | N/A |
| Lack of alternatives | N/A | | | N/A |
| Substantial improvement in life expectancy | N/A | | | No trial available that fulfilled PICOS-criteria |
|  | **Yes** | | **No** | **Notes** |
| Discounting | N/A | | | N/A |
| Different discount rates explored | N/A | | | N/A |
| Uncertainty | X | |  | No evidence |
| Alternative payment models explored | N/A | | | N/A |

**Table 37 Checklist for assessing gene therapies. Drug: darvadstrocel (Alofisel) for treating perianal fistula in Crohn’s disease. Setting: England (NICE)**

| Item | Yes | No | Notes |
| --- | --- | --- | --- |
| Surrogate endpoint used | X |  | **Validation given?** N/A  Primary endpoint was combined remission at week 24.  The authority expressed concerns on the post-hoc nature of the analysis for clinical and patient-centric remission and relapse |
| Rare disease | X |  | **Prevalence**: N/A  Complex perianal fistula(e) was recognized as an orphan disease |
| Serious condition | X |  | High unmet need pointed out by manufacturer, patient organizations and expert opinion and recognized by the authority |
| Single-arm trial |  | X | **Matched historical cohort used?** No, the pivotal trial (ADMIRE-CD) was randomized and double blind. The authority expressed concerns on the lack of a confirmatory study due to the modest effect size (underpowered post-hoc analysis) |
| Pediatric population |  | X | **Age range**: the treatment is indicated for the adult population ( ≥ 18 years of age); median age of onset of the condition is between 15 and 30 years |
| Reporting of adverse consequences and risks | X |  | N/A |
| Size of clinical trial | **Number of patients**: 289 | | N/A |
| Length of clinical trial | **Duration in months**: 22 (96 weeks) | | The authority expressed concerns on long-term outcomes (repeated administration and long-term efficacy and safety) |
| Extrapolation to long-term outcomes | **Duration in months**: 480 (40 years) | | The authority expressed concerns on multiple aspects of the extrapolation to long-term outcomes (possibility of repeated administrations, the manufacturer’s selection of time to relapse and time to remission) |
|  | **Yes** | **No** | **Quantification** |
| Severe disease | X |  | Severity was recognized in terms of improvement from severe condition. The company did not claim that the treatment meets NICE’s end of life criteria |
| Value to caregivers | X |  | No explicit quantification. Detrimental impact of the condition on carers physical and mental health was reported by patient organizations and was recognized by the authority |
| Insurance value |  | X | N/A |
| Scientific spillovers |  | X | N/A |
| Lack of alternatives | X |  | High unmet need pointed out by manufacturer, patient organizations and expert opinion and recognized by the authority |
| Substantial improvement in life expectancy |  | X | The condition does not have a direct impact in decreasing life expectancy |
|  | **Yes** | **No** | **Notes** |
| Discounting | X |  | 3.5% per annum for costs, 1.5% per annum for health outcomes. The authority expressed concerns on the suitability of the choice |
| Different discount rates explored | X |  | Discount rates analyzed in scenario analyses were 0%, 3.5%, and 10% |
| Uncertainty | X |  | PSA, DSA, scenario analyses. The authority proposed 8 sets of exploratory analyses |
| Alternative payment models explored | N/A | | N/A (treatment not recommended) |

**Table 38** **Checklist for assessing gene therapies. Drug: darvadstrocel (Alofisel) for treating perianal fistula in Crohn’s disease. Setting: Scotland (SMC)**

| Item | Yes | No | Notes |
| --- | --- | --- | --- |
| Surrogate endpoint used | X |  | **Validation given?** No  In general, the authority remarked the absence of validated outcomes for assessing the condition |
| Rare disease | X |  | **Prevalence**: N/A  The authority noted the orphan designation by EMA for the treatment and agreed upon it, justifying greater uncertainty in the economic case |
| Serious condition | X |  | High unmet need was pointed out by experts and patient associations; the authority accepted the claim |
| Single-arm trial |  | X | **Matched historical cohort used?** No |
| Pediatric population |  | X | **Age range**: adult population  The authority recognized that the condition is often diagnosed at a young age, generating a lifetime of disease burden |
| Reporting of adverse consequences and risks | X |  | The authority expressed concerns on safety of repeated administration |
| Size of clinical trial | **Number of patients**: 212 | | N/A |
| Length of clinical trial | **Duration in months**: 12 (52 weeks), limited data on 24 (104 weeks) | | The authority expressed concerns on long-term outcomes |
| Extrapolation to long-term outcomes | **Duration in months**: 480 (40 years) | | The authority expressed concerns on the impact of specific parametric assumptions on cost-effectiveness |
|  | **Yes** | **No** | **Quantification** |
| Severe disease | X |  | Not end-of-life condition; however, the authority recognized the severity of the condition (no explicit quantification) |
| Value to caregivers | X |  | No explicit quantification. Detrimental impact of the condition on carers physical and mental health was reported by patient organizations and was recognized by the authority |
| Insurance value | N/A | | N/A |
| Scientific spillovers | N/A | | N/A |
| Lack of alternatives | X |  | No explicit quantification. Lack of alternatives was reported by experts and was recognized by the authority |
| Substantial improvement in life expectancy |  | X | The condition does not have a direct impact in decreasing life expectancy. The authority expressed concerns on the magnitude of the incremental clinical effectiveness for the primary outcome and the lack of statistical significance on the incremental clinical effectiveness of the secondary outcomes |
|  | **Yes** | **No** | **Notes** |
| Discounting | N/A | | N/A |
| Different discount rates explored | N/A | | N/A |
| Uncertainty | X |  | Based on scenario analyses, the authority expressed concerns on the impact of specific parametric assumptions on cost-effectiveness |
| Alternative payment models explored | N/A | | A Patient Access Scheme (PAS) was proposed by the submitting company and was assessed by the Patient Access Scheme Assessment Group (PASAG) as acceptable for implementation. However, the treatment was not recommended because the submitting company did not present a sufficiently robust economic analysis |

**Table 39** **Checklist for assessing gene therapies. Drug: darvadstrocel (Alofisel) for treating perianal fistula in Crohn’s disease. Setting: France (HAS)**

| Item | Yes | | No | Notes |
| --- | --- | --- | --- | --- |
| Surrogate endpoint used | X | |  | **Validation given?** N/A  Primary endpoint is the proportion of patients in remission |
| Rare disease | X | |  | **Prevalence**: 1/1,000 in France  Orphan drug designation |
| Serious condition | X | |  | High unmet need recognized |
| Single-arm trial |  | | X | **Matched historical cohort used?** No  Phase III, randomized, double-blind study. The HTA body emphasized the good methodological quality of the study |
| Pediatric population |  | | X | **Age range**: 20-30 years (at diagnosis) |
| Reporting of adverse consequences and risks | X | |  | The HTA body expressed concerns about long-term safety |
| Size of clinical trial | **Number of patients**: 205 (212 ITT) | | | N/A |
| Length of clinical trial | **Duration in months**: 24 | | | N/A |
| Extrapolation to long-term outcomes | **Duration in months**: N/A | | | N/A |
|  | **Yes** | | **No** | **Quantification** |
| Severe disease | X | |  | No explicit quantification, the HTA body defined the disease as “severe and disabling” and recognized significant morbidity and loss in quality of life |
| Value to caregivers | N/A | | | N/A |
| Insurance value | N/A | | | N/A |
| Scientific spillovers | N/A | | | N/A |
| Lack of alternatives |  | | X | Alternatives exist; however, they are not curative. |
| Substantial improvement in life expectancy |  | X | | Extending life expectancy is not the objective of the treatment |
|  | **Yes** | | **No** | **Notes** |
| Discounting | N/A | | | N/A |
| Different discount rates explored | N/A | | | N/A |
| Uncertainty | N/A | | | N/A |
| Alternative payment models explored | N/A | | | N/A |

**Table 40 Checklist for assessing gene therapies. Drug: darvadstrocel (Alofisel) for treating perianal fistula in Crohn’s disease. Setting: Germany (G-BA)**

| Item | Yes | No | Notes |
| --- | --- | --- | --- |
| Surrogate endpoint used |  | X | **Validation given?** N/A |
| Rare disease |  | X | **Prevalence**: 90 - 230 patients  Orphan drug designation |
| Serious condition | N/A | | N/A |
| Single-arm trial |  | X | **Matched historical cohort used?** N/A |
| Pediatric population |  | X | **Age range**: adult patients |
| Reporting of adverse consequences and risks | X |  | Reporting is standard. |
| Size of clinical trial | **Number of patients**: 212 | |  |
| Length of clinical trial | **Duration in months**: 12 | |  |
| Extrapolation to long-term outcomes | N/A | | N/A |
|  | **Yes** | **No** | **Quantification** |
| Severe disease | N/A | | N/A |
| Value to caregivers | N/A | | N/A |
| Insurance value | N/A | | N/A |
| Scientific spillovers | N/A | | N/A |
| Lack of alternatives | N/A | | N/A |
| Substantial improvement in life expectancy |  | X | No deaths |
|  | **Yes** | **No** | **Notes** |
| Discounting | N/A | | N/A |
| Different discount rates explored | N/A | | N/A |
| Uncertainty | X |  | Methodological limitations; added benefit not quantifiable |
| Alternative payment models explored | N/A | | N/A |

**Table 41** **Checklist for assessing gene therapies. Drug: sipuleucel-T (Provenge) for treating asymptomatic or minimally symptomatic metastatic hormone-relapsed prostate cancer. Setting: England (NICE)**

| Item | Yes | No | Notes |
| --- | --- | --- | --- |
| Surrogate endpoint used |  | X | **Validation given?** N/A  The primary endpoint of the pivotal trial (IMPACT) was overall survival |
| Rare disease | N/A | | **Prevalence**: N/A |
| Serious condition | N/A | | N/A |
| Single-arm trial |  | X | **Matched historical cohort used?** No  The pivotal trial was placebo-controlled; the manufacturer produced indirect treatment comparisons on which the authority expressed major concerns |
| Pediatric population |  | X | **Age range**: it was deducible that the treatment addresses the adult population |
| Reporting of adverse consequences and risks | X |  | The authority expressed concerns on the inability to establish the adverse-event profile of the treatment compared with only best supportive care |
| Size of clinical trial | **Number of patients**: 512 | | N/A |
| Length of clinical trial | **Duration in months**: 34 (median follow-up) | | N/A |
| Extrapolation to long-term outcomes | **Duration in months**: 120 (10 years) | | The manufacturer presented parametric extrapolations on which the authority expressed major concerns |
|  | **Yes** | **No** | **Quantification** |
| Severe disease | N/A | | The condition does not satisfy NICE end-of-life criteria |
| Value to caregivers | N/A | | N/A |
| Insurance value |  | X | No other dimensions of value not captured in QALYs were deemed relevant |
| Scientific spillovers |  | X | No other dimensions of value not captured in QALYs were deemed relevant |
| Lack of alternatives | N/A | | N/A |
| Substantial improvement in life expectancy | N/A | | Incremental QALY gain of 0.354 in base-case analysis. Patient organizations claimed that the most important benefits of the treatment was its potential to extend life |
|  | **Yes** | **No** | **Notes** |
| Discounting | X |  | Both costs and utilities were discounted at 3.5% per year |
| Different discount rates explored | N/A | | N/A |
| Uncertainty | X |  | The company performed subgroup analyses, sensitivity and scenario analyses (different hazard rations for indirect comparison, different utility values, different parametric distribution for overall survival, different assumptions on docetaxel usage. Further scenario analyses were requested by the evidence review group |
| Alternative payment models explored | N/A | | The treatment is not recommended |

**Table 42 Checklist for assessing gene therapies. Drug: sipuleucel-T (Provenge) for treating asymptomatic or minimally symptomatic metastatic hormone-relapsed prostate cancer. Setting: Germany (G-BA)**

| Item | Yes | No | Notes |
| --- | --- | --- | --- |
| Surrogate endpoint used |  | X | **Validation given?** N/A |
| Rare disease |  | X | **Prevalence**: 11,690 - 24,480 patients  No orphan drug designation |
| Serious condition | N/A | | N/A |
| Single-arm trial |  | X | **Matched historical cohort used?** N/A |
| Pediatric population |  | X | **Age range**: adult patients |
| Reporting of adverse consequences and risks | X |  | Reporting is standard. |
| Size of clinical trial | **Number of patients**: IMPACT: 512  D9901: 127  D9902A: 110 | |  |
| Length of clinical trial | **Duration in months**: IMPACT: 20.6 (intervention) / 19.3 (placebo)  D9901: not provided  D9902A: not provided | |  |
| Extrapolation to long-term outcomes | N/A | | N/A |
|  | **Yes** | **No** | **Quantification** |
| Severe disease | N/A | | N/A |
| Value to caregivers | N/A | | N/A |
| Insurance value | N/A | | N/A |
| Scientific spillovers | N/A | | N/A |
| Lack of alternatives | N/A | | N/A |
| Substantial improvement in life expectancy |  | X | Not quantifiable due to the high bias potential and the uncertainties |
|  | **Yes** | **No** | **Notes** |
| Discounting | N/A | | N/A |
| Different discount rates explored | N/A | | N/A |
| Uncertainty | X |  | Hint |
| Alternative payment models explored | N/A | | N/A |

**Table 43 Checklist for assessing gene therapies. Drug: alipogene tiparvovec (Glybera) for treating adults with lipoprotein lipase deficiency who have severe or multiple attacks of pancreatitis (inflammation of the pancreas) despite maintaining a low-fat diet.**

**Setting: France (HAS)**

| Item | Yes | No | Notes |
| --- | --- | --- | --- |
| Surrogate endpoint used | X |  | **Validation given?** N/A  The primary endpoint is the percentage of patients experiencing a median reduction of a specific indicator |
| Rare disease | X |  | **Prevalence**: N/A  Orphan drug designation, the HTA body defined the disease as “rare and inherited” |
| Serious condition | N/A | | N/A |
| Single-arm trial | X |  | **Matched historical cohort used?** N/A  The HTA body expressed concerns about the study design (prospective, open-label, non-comparative |
| Pediatric population |  | X | **Age range**: adult patients |
| Reporting of adverse consequences and risks | X |  | The HTA body expressed concerns about lack of long-term safety data |
| Size of clinical trial | **Number of patients**: 27 | | The HTA body expressed concerns about the sample size |
| Length of clinical trial | **Duration in months**: <12 | | The HTA body expressed concerns about the limited follow-up |
| Extrapolation to long-term outcomes | **Duration in months**: N/A | | N/A |
|  | **Yes** | **No** | **Quantification** |
| Severe disease | X |  | No explicit quantification, high morbidity and worsening in quality of life recognized |
| Value to caregivers | N/A | | N/A |
| Insurance value | N/A | | N/A |
| Scientific spillovers | N/A | | N/A |
| Lack of alternatives | X |  | No clinically meaningful comparators existing |
| Substantial improvement in life expectancy |  | X | Modest effect on pancreatitis and triglyceride levels not maintained below 1 year |
|  | **Yes** | **No** | **Notes** |
| Discounting | N/A | | N/A |
| Different discount rates explored | N/A | | N/A |
| Uncertainty | N/A | | N/A |
| Alternative payment models explored | N/A | | N/A |

**Table 44 Checklist for assessing gene therapies. Drug: : alipogene tiparvovec (Glybera) for treating adults with lipoprotein lipase deficiency who have severe or multiple attacks of pancreatitis (inflammation of the pancreas) despite maintaining a low-fat diet. Setting: Germany (G-BA)**

| Item | Yes | No | Notes |
| --- | --- | --- | --- |
| Surrogate endpoint used |  | X | **Validation given?** N/A |
| Rare disease | X |  | **Prevalence**: 17 - 35 patients  Orphan drug designation |
| Serious condition | N/A | | N/A |
| Single-arm trial | X |  | **Matched historical cohort used?** The manufacturer submitted various non-randomized and not-controlled studies and retrospective data reviews but no indirect comparisons. |
| Pediatric population |  | X | **Age range**: adult patients |
| Reporting of adverse consequences and risks |  | X | No trial available that fulfilled PICOS-criteria |
| Size of clinical trial | N/A | | No trial available that fulfilled PICOS-criteria |
| Length of clinical trial | N/A | | No trial available that fulfilled PICOS-criteria |
| Extrapolation to long-term outcomes | N/A | | N/A |
|  | **Yes** | **No** | **Quantification** |
| Severe disease | N/A | | N/A |
| Value to caregivers | N/A | | N/A |
| Insurance value | N/A | | N/A |
| Scientific spillovers | N/A | | N/A |
| Lack of alternatives | N/A | | N/A |
| Substantial improvement in life expectancy |  | X | Mortality data was not collected |
|  | **Yes** | **No** | **Notes** |
| Discounting | N/A | | N/A |
| Different discount rates explored | N/A | | N/A |
| Uncertainty | X |  | Hint |
| Alternative payment models explored | N/A | | N/A |

**Table 45** **Checklist for assessing gene therapies. Drug: onasemnogene abeparvovec (Zolgensma) for treating spinal muscular atrophy**

**Setting: England (NICE)**

| Item | Yes | No | Notes |
| --- | --- | --- | --- |
| Surrogate endpoint used | X |  | **Validation given?** N/A  Time to death or requirement of respiratory assistance (considered a surrogate for death) are the secondary endpoints of the phase I/IIa study (i.e., the only one which was completed at the time of the submission). |
| Rare disease | X |  | **Prevalence**: from 0.04 to 0.28 per 100,000 population.  The authority recognized the condition as rare. |
| Serious condition | X |  | Unmet need was claimed by the manufacturer and clinical experts (while patient organizations did not seem to perceive it), it was recognized by the authority. It was recognized as a life-shortening condition. |
| Single-arm trial | X |  | **Matched historical cohort used?** Yes. The authority expressed concerns on the generalizability of results to the UK because the historical cohorts enrolled patients primarily in the US where clinical practice is different. The other main concern was on the possibility to perform only naïve comparisons due to the small sample size. |
| Pediatric population | X |  | **Age range**: N/A  Onset of the condition takes place before 6 months of age. |
| Reporting of adverse consequences and risks | X |  | N/A |
| Size of clinical trial | **Number of patients**: 15 | | It refers to the sample size of the phase I/IIa study. Ongoing phase III studies enrolled 33, 22 and 29 patients. The authority expressed concerns on the small sample size. |
| Length of clinical trial | **Duration in months**: 24 | | The authority expressed concerns on the lack of long-term efficacy and safety data. |
| Extrapolation to long-term outcomes | **Duration in months**: lifetime horizon | | Parametric extrapolation of survival data. |
|  | **Yes** | **No** | **Quantification** |
| Severe disease | X |  | The authority recognized the severity of the disease and its fatality if treated with best supportive care. |
| Value to caregivers | X |  | No explicit quantification. The manufacturer and patient organizations claimed a significant burden on caregivers and that the treatment, by improving infants’ autonomy, could increase the likelihood of labor market participation for parents. The authority did not dispute the claim. |
| Insurance value | N/A | | N/A |
| Scientific spillovers | X |  | No explicit quantification. The manufacturer claimed the treatment will lead to greater understanding of epidemiology, pathology and management of the condition and opportunities for treatment optimization. The authority did not dispute the claim. |
| Lack of alternatives |  | X | Nusinersen is an alternative; it was underlined that it does not represent established clinical practice in the UK yet. |
| Substantial improvement in life expectancy | X |  | Phase I/IIa study results show that all participants were alive and event-free at 24 months. |
|  | **Yes** | **No** | **Notes** |
| Discounting | X |  | 3.5% for costs. |
| Different discount rates explored | X |  | N/A |
| Uncertainty | X |  | Deterministic, probabilistic, and scenario-based sensitivity analyses. |
| Alternative payment models explored | N/A | | The manufacturer proposed a confidential simple discount on the published UK list price. |

**Table 46 Checklist for assessing gene therapies. Drug: onasemnogene abeparvovec (Zolgensma) for treating spinal muscular atrophy**

**Setting: Scotland (SMC)**

| Item | Yes | No | Notes |
| --- | --- | --- | --- |
| Surrogate endpoint used | X |  | **Validation given?** N/A  Primary endpoints: sitting independently for at least 30 seconds at 18 months, survival at 14 months (defined by the avoidance of either death or requiring permanent ventilatory support). Secondary outcomes: proportion of patients maintaining the ability to thrive at 18 months of age and the proportion of patients who were independent of ventilatory support at 18 months of age. |
| Rare disease | X |  | **Prevalence**: N/A  The condition meets SMC orphan criteria, the treatment was assessed under the orphan medicines process. |
| Serious condition | X |  | Life shortening condition (patients are unlikely to survive 2 years of age), unmet need recognized. |
| Single-arm trial | X |  | **Matched historical cohort used?** Yes  The authority expressed concerns on the lack of direct comparability with another currently available treatment (nusinersen) and the lack of randomization. |
| Pediatric population | X |  | **Age range**: N/A |
| Reporting of adverse consequences and risks | X |  | The authority expressed concerns on the fact that safety data were available only for a small number of patients and the lack of long-term safety data. |
| Size of clinical trial | **Number of patients**:  22 (STR1VE US)  15 (START)  13 (LT-001)  29 (SPR1NT) | | The authority expressed concerns for the small number of patients included. |
| Length of clinical trial | **Duration in months**:  18 (STR1VE US)  24 (START)  53 (4.4 years LT-001) | | The authority expressed concerns on the paucity of long-term data. |
| Extrapolation to long-term outcomes | **Duration in months**: N/A  Lifetime horizon | | The authority expressed concerns on validating assumptions regarding the choices of extrapolation distributions used and assumptions surrounding longer term treatment benefit. |
|  | **Yes** | **No** | **Quantification** |
| Severe disease | X |  | Improvement from severe condition was recognized (e.g., patients with SMA Type 1 rapidly lose motor and respiratory function leading to death, usually before 2 years of age). |
| Value to caregivers | X |  | No explicit quantification, it was recognized that the treatment could potentially reduce care requirements and remove the need for frequent hospital visits. Patient associations appreciated the single-infusion formulation. |
| Insurance value | N/A | | N/A |
| Scientific spillovers | N/A | | N/A |
| Lack of alternatives | X |  | The existing alternative (nusinersen) is restricted to patients with symptomatic SMA type 1 and its lifelong administration was deemed challenging. |
| Substantial improvement in life expectancy | X |  | The authority recognized significant difference in survival rates. |
|  | **Yes** | **No** | **Notes** |
| Discounting | X |  | N/A |
| Different discount rates explored | X |  | N/A |
| Uncertainty | X |  | The choice of distributions for long-term extrapolation was tested in sensitivity analysis; scenario analyses were performed. The authority expressed concerns on the uncertainty surrounding utility values. |
| Alternative payment models explored |  | X | The authority deemed acceptable the patient access scheme proposed by the manufacturer, based on a confidential discount on the list price (£ 1,795,000 per course). The authority expressed concerns on the service implications and financial risk associated with the high upfront cost. |

**Table 47 Checklist for assessing gene therapies. Drug: onasemnogene abeparvovec (Zolgensma) for treating spinal muscular atrophy**

**Setting: United States (ICER)**

| Item | Yes | No | Notes |
| --- | --- | --- | --- |
| Surrogate endpoint used |  | X | **Validation given?** N/A  Mortality is the first outcome of interest in the analysis. |
| Rare disease | X |  | **Prevalence**: N/A  Incidence: approximately one in 10,000 live births or about 500 new cases per year. The eligible patient population is estimated at fewer than approximately 10,000 individuals |
| Serious condition | X |  | High unmet need and limited life expectancy (historically < 2 years, more recently up to 4 years) were recognized |
| Single-arm trial | X |  | **Matched historical cohort used?** No, in the economic model, in order to compare Zolgensma to best supportive care, the sham arm in ENDEAR was used to inform the comparison. The HTA body expressed concerns on using historical controls because they can exaggerate perceived treatment effects due to standard of care improvements and variable natural history of the disease. |
| Pediatric population | X |  | **Age range**: in the economic model mean age considered was 4.4 months for infantile-onset (type I) SMA, 2 years for later onset (type II/III) SMA, 21 days for presymptomatic SMA |
| Reporting of adverse consequences and risks | X |  | The HTA body expressed concerns about the lack of data on long-term safety, especially in terms of liver function |
| Size of clinical trial | **Number of patients**: 15 | | The HTA body expressed concerns about the generalizability of results to the wider population of patients with SMA due to narrow eligibility criteria of trials and limited sample size (unselected patients are more severely ill, experience more comorbidities [e.g., scoliosis], or have a different genetic profile) |
| Length of clinical trial | **Duration in months**:  mean post-treatment age in START: 39 months. The analysis is based on the data cut-off at August 7, 2017; 10/15 patients in CL-101 had 24 months follow-up | | The HTA body expressed concerns about the long-term durability of the treatment (unclear treatment pathway in case of gene expression waning, antibodies formation, usage in combination with Spinraza) |
| Extrapolation to long-term outcomes | **Duration in months**: N/A (“lifetime”) | | Parametric extrapolation; the de novo economic model used motor function milestones to define broad health states and assumed relationships between motor function milestone-based health states and survival. |
|  | **Yes** | **No** | **Quantification** |
| Severe disease | X |  | It was recognized that the intervention addresses a condition of particularly high severity in terms of impact on length of life (historically < 2 years, more recently up to 4 years) |
| Value to caregivers | X |  | No explicit quantification, it was recognized that the intervention can significantly reduce caregiver or broader family burden |
| Insurance value | N/A | |  |
| Scientific spillovers | N/A | |  |
| Lack of alternatives |  | X | A disease-modifying therapy was approved (Spinraza); however, it was recognized important uncertainties remain regarding the effectiveness of Spinraza in certain patient subgroups, its duration of benefit, and the comparative effectiveness with respect to Zolgensma |
| Substantial improvement in life expectancy | X |  | All infants in the Phase I CL-101 trial were alive following at least 24 months of follow-up; the HTA body recognized that Zolgensma provides a substantial net health benefit |
|  | **Yes** | **No** | **Notes** |
| Discounting | X |  | 3% per annum |
| Different discount rates explored | X |  | 1.5% per annum |
| Uncertainty | X |  | Scenario analyses: taking a modified societal perspective, alternative survival, cost, and utility assumptions |
| Alternative payment models explored | N/A | | At the time of the analysis Zolgensma was not approved by FDA yet |

**Table 48 Checklist for assessing gene therapies. Drug: onasemnogene abeparvovec (Zolgensma) for treating spinal muscular atrophy**

**Setting: Canada (CADTH)**

| Item | Yes | No | Notes |
| --- | --- | --- | --- |
| Surrogate endpoint used |  | X | **Validation given?** N/A  Survival (i.e., avoidance of death or permanent ventilation) at 14 months of age is the co-primary endpoint of pivotal phase III study |
| Rare disease | X |  | **Prevalence**: N/A |
| Serious condition | X |  | Life-shortening condition and unmet need recognized. Another treatment exists (nusinersen) but patients and clinicians are concerned about the potential harm of administering it intrathecally every 3 months |
| Single-arm trial | X |  | **Matched historical cohort used?** Yes, using selected patients from the Pediatric Neuromuscular Clinical Research Network dataset. The HTA body expressed concerns about using a matched historical cohort because of lack of statistical adjustments for differences in patient characteristics, collateral treatments, and observation time frames. The HTA body expressed concerns also on the results of indirect treatment comparisons with nusinersen because of differences in study design, entry criteria, patient characteristics, and outcome definitions |
| Pediatric population | X |  | **Age range**: N/A  A treatment initiation criterion is being 180 days of age or younger |
| Reporting of adverse consequences and risks | X |  | The HTA body expressed concerns about liver injury, increased troponin levels, and thrombocytopenia |
| Size of clinical trial | **Number of patients**: 22 (STR1VE-US), 30 (SPR1NT) | | N/A |
| Length of clinical trial | **Duration in months**: 18 (STR1VE-US) | | The HTA body expressed concerns about the limited time horizon of 18 months for a life-long condition |
| Extrapolation to long-term outcomes | **Duration in months**: 924 (77 years) | | The HTA body expressed concerns about the long-term efficacy of the treatment and the fact that extrapolation of clinical benefit was based on assumptions that could not be tested |
|  | **Yes** | **No** | **Quantification** |
| Severe disease | X |  | No explicit quantification, the severity of the condition was identified by the difficulty breathing, swallowing and loss of mobility, loss of patient independence, increased load on families, negative impact on mental and emotional well-being |
| Value to caregivers | X |  | No explicit quantification, potential for reduction of the burden on caregivers |
| Insurance value | N/A | | N/A |
| Scientific spillovers | N/A | | N/A |
| Lack of alternatives |  | X | An alternative treatment exists (nusinersen); however, patients and parents expressed concerns about the trimestral intrathecal administration (painful, invasive, and challenging for young children) |
| Substantial improvement in life expectancy | X |  | 90% were alive without permanent ventilation at 14 months of age in STR1VE-US; all infants were alive without permanent ventilation (event-free survival) at 14 months in SPR1NT (interim analysis) |
|  | **Yes** | **No** | **Notes** |
| Discounting | N/A | | N/A |
| Different discount rates explored | N/A | | N/A |
| Uncertainty | X |  | Scenario analyses (e.g., different patient population). The HTA body expressed concerns about the estimated cost-effectiveness because most of the modelled benefits are accrued in time periods beyond which any clinical data are available |
| Alternative payment models explored |  | X | Pricing conditions feature “a reduction in price” (at least 90% to achieve an ICER below $50,000 per QALY gained) |

**Table 49**  **Checklist for assessing gene therapies. Drug: onasemnogene abeparvovec (Zolgensma) for treating spinal muscular atrophy**

**Setting: Italy (AIFA)**

| Item | Yes | | No | Notes |
| --- | --- | --- | --- | --- |
| Surrogate endpoint used |  | | X | **Validation given?** N/A  Co-primary endpoint was survival without need for mechanical ventilation at 14 months |
| Rare disease | X | |  | **Prevalence**: 1:40-1:60 individuals in the general population are carriers of mutations in SMN1 that can cause the disease |
| Serious condition | X | |  | High unmet clinical need recognized; the HTA body acknowledged the existence of an alternative treatment that produces clinically relevant outcomes (significant although limited) but claimed it is an invasive life-long treatment with unsatisfactory toxicity profile. Overall, the HTA body recognized a moderate therapeutic need. |
| Single-arm trial | X | |  | **Matched historical cohort used?** Yes, PNCR and Neuronext studies.  The HTA body judged the quality of evidence very low because of the design of the pivotal studies (open label, single-arm) and applied a downgrading for lack of indirectness. |
| Pediatric population | X | |  | **Age range**: N/A  Onset before 6 months of age |
| Reporting of adverse consequences and risks | X | |  | The HTA body expressed concerns about possible long-term adverse consequences (i.e., cardiac toxicity, carcinogenicity, toxicity to dorsal root ganglia |
| Size of clinical trial | **Number of patients**:  22 (STR1VE-US) | | | The HTA body expressed concerns about the limited size of the pivotal studies |
| Length of clinical trial | **Duration in months**: N/A | | | N/A |
| Extrapolation to long-term outcomes | **Duration in months**: lifetime | | | N/A |
|  | **Yes** | | **No** | **Quantification** |
| Severe disease | X |  | | Median survival without mechanical ventilation is 10.5 months, 8% of untreated patients survive to the 20^th^ month |
| Value to caregivers | N/A | | | N/A |
| Insurance value | N/A | | | N/A |
| Scientific spillovers | N/A | | | N/A |
| Lack of alternatives |  | | X | The HTA body acknowledged the existence of an alternative treatment that produces clinically relevant outcomes (significant although limited) but claimed it is an invasive life-long treatment with unsatisfactory toxicity profile. |
| Substantial improvement in life expectancy | X | |  | The HTA judged the therapeutic added value important based on event-free survival data |
|  | **Yes** | | **No** | **Notes** |
| Discounting | X |  | | 3% per annum applied to costs and outcomes |
| Different discount rates explored | X |  | | 1.5% |
| Uncertainty | X |  | | DSA, PSA, scenario analysis |
| Alternative payment models explored | X |  | | Payment at results at delivery, at 12, 24, 36, and 48 months |

**Table 50** **Checklist for assessing gene therapies. Drug: onasemnogene abeparvovec (Zolgensma) for treating spinal muscular atrophy**

**Setting: France (HAS)**

| Item | Yes | No | Notes |
| --- | --- | --- | --- |
| Surrogate endpoint used |  | X | **Validation given?** N/A  The primary endpoint considered is survival without permanent ventilation |
| Rare disease | X |  | **Prevalence**: 20/100,000 births globally  The treatment received orphan drug designation |
| Serious condition | X |  | High unmet need recognized, limited survival (10.5-13.5 months of median survival without permanent ventilation) |
| Single-arm trial | X |  | **Matched historical cohort used?** Yes.  The HTA body expressed concerns about the lack of direct comparison (not only with the active comparator Spinraza but also with the natural evolution of the disease) and cautioned about the methodological limitations of the MAIC |
| Pediatric population | X |  | **Age range**: N/A  Mean age 3.5 months |
| Reporting of adverse consequences and risks | X |  | The HTA body expressed concerns about possible severe hepatic adverse events and lack of long-term safety data |
| Size of clinical trial | **Number of patients**:  22 (STR1VE US)  15 (START) | | The HTA body deemed the sample size too small to generalize results to a larger population of patients suffering from SMA |
| Length of clinical trial | **Duration in months**: 24 | | The HTA expressed concerns about long-term effectiveness |
| Extrapolation to long-term outcomes | **Duration in months**: 120 (10 years) | | The manufacturer produced parametric extrapolation guided by AIC and BIC criteria. The HTA body expressed concerns about OS extrapolation |
|  | **Yes** | **No** | **Quantification** |
| Severe disease | X |  | Limited survival (10.5-13.5 months of median survival without permanent ventilation). The HTA body claimed that it is a severe disease impacting survival, especially type I and II |
| Value to caregivers | X |  | The disease has a strong impact on caregiver’s quality of life and the need for alternative treatments has been emphasized by patient associations |
| Insurance value | N/A | |  |
| Scientific spillovers | N/A | |  |
| Lack of alternatives |  | X | However, the HTA body claimed the existence of a need for more effective alternatives in the long-term, especially for type I and II |
| Substantial improvement in life expectancy | X |  | 100% of patients alive without the need for mechanical ventilation after 24 moths |
|  | **Yes** | **No** | **Notes** |
| Discounting | X |  | 2.5% |
| Different discount rates explored | X |  | 0%, 4% |
| Uncertainty | X |  | Scenario analysis, DSA, PSA |
| Alternative payment models explored | N/A | | N/A |

**Table 51 Checklist for assessing gene therapies. Drug: onasemnogene abeparvovec (Zolgensma) for treating spinal muscular atrophy. Setting: Germany (G-BA)**

| Item | Yes | | No | Notes |
| --- | --- | --- | --- | --- |
| Surrogate endpoint used |  | | X | **Validation given?** N/A |
| Rare disease | X | |  | **Prevalence**: 45 - 65 patients (5q-SMA type 3 and up to 3 copies of the SMN 2 gene)  Orphan drug designation |
| Serious condition | N/A | | | N/A |
| Single-arm trial | X | |  | **Matched historical cohort used?** The presented comparisons of individual arms from different studies between onasemnogen-abeparvovec and nusinersen are unsuitable for the benefit assessment of onasemnogen-abeparvovec due to the large uncertainties and accordingly cannot be used to derive an added benefit. |
| Pediatric population | X | |  | **Age range**: N/A |
| Reporting of adverse consequences and risks |  | | X | Not reported due to large uncertainties in the data basis |
| Size of clinical trial | **Number of patients**:  15 (START)  33 (STR1VE-EU)  22 (STR1VE-US) | | | N/A |
| Length of clinical trial | **Duration in months**:  12-25 | | | Observation times for individual patients |
| Extrapolation to long-term outcomes | N/A | | | N/A |
|  | **Yes** | | **No** | **Quantification** |
| Severe disease | N/A | | | N/A |
| Value to caregivers | N/A | | | N/A |
| Insurance value | N/A | | | N/A |
| Scientific spillovers | N/A | | | N/A |
| Lack of alternatives | N/A | | | N/A |
| Substantial improvement in life expectancy |  | X | | Large uncertainties in the data basis. However, taking into account the available evidence on the medical benefit of onasemnogen abeparvovec, the severity of the disease and the statements of the medical societies on the current reality of care, onasemnogen abeparvovec may represent a relevant treatment option. |
|  | **Yes** | | **No** | **Notes** |
| Discounting | N/A | | | N/A |
| Different discount rates explored | N/A | | | N/A |
| Uncertainty | X | |  | Large uncertainty |
| Alternative payment models explored | N/A | | | Comprehensive quality assurance measures (prerequisites for the provision of therapy) have been specified. |

**Information Sources: Spain**

Kymriah DLBCL - <https://www.aemps.gob.es/medicamentosUsoHumano/informesPublicos/docs/IPT-tisagenlecleucel-kymriah-LAL-LCGB.pdf?x90532>

Kymriah ALL

https://www.aemps.gob.es/medicamentosUsoHumano/informesPublicos/docs/IPT-tisagenlecleucel-kymriah-LAL-LCGB.pdf?x90532

Yescarta

https://www.aemps.gob.es/medicamentosUsoHumano/informesPublicos/docs/IPT-axicabtagen-ciloleucel-Yescarta-LDCGB-LPMCGB.pdf?x90532

Luxturna –

https://www.aemps.gob.es/informa/informes-de-posicionamiento-terapeutico/informe-de-posicionamiento-terapeutico-de-voretigen-neparvovec-luxturna-para-distrofia-retiniana-asociada-a-la-mutacion-rpe65-bialelica/

Strimvelis - No IPT – No Genesis Group report

Imlygic- No IPT – No Genesis Group report

https://www.aemps.gob.es/medicamentosUsoHumano/informesPublicos/docs/IPT-talimogen-laherparepvec-Imlygic-melanoma.pdf?x90532

Alofisel

https://www.aemps.gob.es/medicamentosUsoHumano/informesPublicos/docs/IPT-darvadstrocel-Alofisel.pdf?x90532

Provenge – No IPT – No Genesis Group report

Glybera - No IPT – No Genesis Group report

Zolgensma - No IPT – No Genesis Group report

**Table 52 Checklist for assessing gene therapies. Drug: tisagenlecleucel-T (Kymriah) for treating relapsed or refractory diffuse large b-cell lymphoma. Setting: Spain – National Level – IPT (Therapeutic Positioning Report)**

| Item | Yes | No | Notes |
| --- | --- | --- | --- |
| Surrogate endpoint used | X |  | **Validation given?** N/A  Global response rate was the primary endpoint. Overall survival was a secondary endpoint |
| Rare disease |  | | **Prevalence**:  The assessment included data on incidence only. |
| Serious condition | X |  | No treatment for R/R patients (only palliative care). |
| Single-arm trial | X |  | **Matched historical cohort used?** SCHOLAR-1, CORAL y PIX301 |
| Pediatric population |  | X | **Age range**: 22-76 |
| Reporting of adverse consequences and risks | X |  |  |
| Size of clinical trial | **Number of patients**: 165  (C2201) | | Patients with infusions 93 |
| Length of clinical trial | **Duration in months**: | |  |
| Extrapolation to long-term outcomes | **Duration in months**: | |  |
|  | **Yes** | **No** | **Quantification** |
| Severe disease | X |  | Lack of alternatives y high mortality rate |
| Value to caregivers | N/A | | N/A |
| Insurance value | N/A | | N/A |
| Scientific spillovers | N/A | | N/A |
| Lack of alternatives | X |  | For R/R patients |
| Substantial improvement in life expectancy | X |  |  |
|  | **Yes** | **No** | **Notes** |
| Discounting |  | X |  |
| Different discount rates explored |  | X |  |
| Uncertainty | X |  |  |
| Alternative payment models explored | X |  | It has been implemented and outcomes based agreement for this medicine, but it was not a proposal from the IPT |

**Table 53 Checklist for assessing gene therapies. Drug: tisagenlecleucel-T (Kymriah) for treating relapsed or refractory b-cell acute lymphoblastic leukaemia in people aged up to 25 years . Setting: Spain – National Level – IPT (Therapeutic Positioning Report)**

| Item | Yes | No | Notes |
| --- | --- | --- | --- |
| Surrogate endpoint used | X |  | **Validation given?** N/A  Global response rate was the primary endpoint. Overall survival was a secondary endpoint |
| Rare disease | N/A | | **Prevalence**: N/A  The report included only incidence data |
| Serious condition | X |  | Mortality and curation rate was provided. |
| Single-arm trial | X |  | **Matched historical cohort used?** Yes |
| Pediatric population |  | X | **Age range**: 22-76 |
| Reporting of adverse consequences and risks | X |  | 95% of the patients has any adverse events |
| Size of clinical trial | **Number of patients**: 92  (B2202 study) | | 75 with infusions  Some other trials/patients were considered as B2205J y B2102J |
| Length of clinical trial | **Duration in months**: | | . |
| Extrapolation to long-term outcomes | **Duration in months**: | |  |
|  | **Yes** | **No** | **Quantification** |
| Severe disease | X |  |  |
| Value to caregivers | N/A | | N/A |
| Insurance value | N/A | | N/A |
| Scientific spillovers | N/A | | N/A |
| Lack of alternatives |  | X |  |
| Substantial improvement in life expectancy | X |  |  |
|  | **Yes** | **No** | **Notes** |
| Discounting |  | X |  |
| Different discount rates explored |  | X |  |
| Uncertainty |  | X |  |
| Alternative payment models explored | X |  | It has been implemented and outcomes based agreement for this medicine, but it was not a proposal from the IPT |

**Table 54 Checklist for assessing gene therapies. Drug: axicabtagene ciloleucel (Yescarta) for treating diffuse large b-cell lymphoma and primary mediastinal b-cell lymphoma after 2 or more systemic therapies. Setting: Spain – National Level – IPT (Therapeutic Positioning Report)**

| Item | Yes | No | Notes |
| --- | --- | --- | --- |
| Surrogate endpoint used | X |  | **Validation given?** N/A  Global response rate was the primary endpoint. Overall survival was a secondary endpoint. The IPT agreed with the primary endpoint |
| Rare disease | N/A | | **Prevalence**: N/A  The report included only incidence data |
| Serious condition | X |  | Curable disease, but with bad prognosis for R/R patients |
| Single-arm trial | X |  | **Matched historical cohort used?** Yes |
| Pediatric population |  | X | **Age range**: 23-76 |
| Reporting of adverse consequences and risks | X |  | 100% of the patients has any adverse events |
| Size of clinical trial | **Number of patients**: 111  (ZUMA-1) | | 101 Patients treated |
| Length of clinical trial | **Duration in months**: | | . |
| Extrapolation to long-term outcomes | **Duration in months**: | |  |
|  | **Yes** | **No** | **Quantification** |
| Severe disease | X |  | Arguments provided by manufacturer (progression rate and prognosis compared to other NHL) and patient organizations (impact on physical conditions and quality of life). |
| Value to caregivers | N/A | | N/A |
| Insurance value | N/A | | N/A |
| Scientific spillovers | N/A | | N/A |
| Lack of alternatives |  | X |  |
| Substantial improvement in life expectancy | X |  |  |
|  | **Yes** | **No** | **Notes** |
| Discounting |  | X |  |
| Different discount rates explored |  | X |  |
| Uncertainty |  | X |  |
| Alternative payment models explored | X |  | It has been implemented and outcomes based agreement for this medicine, but it was not a proposal from the IPT |

**Table 55 Checklist for assessing gene therapies. Drug: darvadstrocel (Alofisel) for treating perianal fistula in Crohn’s disease**

**Setting: Spain – National Level – IPT (Therapeutic Positioning Report)**

| Item | Yes | No | Notes |
| --- | --- | --- | --- |
| Surrogate endpoint used | X |  | **Validation given?** N/A  Primary endpoint was combined remission at week 24 and 52. |
| Rare disease |  |  | **Prevalence**: N/A |
| Serious condition | X |  | The treatment of the disease is complex |
| Single-arm trial |  | X | **Matched historical cohort used?** No, the pivotal trial (ADMIRE-CD) was randomized and double blind. |
| Pediatric population |  | X | **Age range**: the treatment is indicated for the adult population ( ≥ 18 years of age); median age of onset of the condition is between 15 and 30 years |
| Reporting of adverse consequences and risks | X |  | Detailed data about the adverse events in the IPT. Concerned about the limited data |
| Size of clinical trial | **Number of patients**: 289  (Cx601-0302 – ADMIRE-CD) | | N/A |
| Length of clinical trial | **Duration in months**: 24 (follow – up 104)) | |  |
| Extrapolation to long-term outcomes | **Duration in months**: | | N/A |
|  | **Yes** | **No** | **Quantification** |
| Severe disease | X |  |  |
| Value to caregivers |  | N |  |
| Insurance value |  | X | N/A |
| Scientific spillovers |  | X | N/A |
| Lack of alternatives |  | X | Darvadstrocel |
| Substantial improvement in life expectancy |  | X | The condition does not have a direct impact in decreasing life expectancy |
|  | **Yes** | **No** | **Notes** |
| Discounting |  | X |  |
| Different discount rates explored |  | X |  |
| Uncertainty |  | X |  |
| Alternative payment models explored | N/A | | It has been implemented and outcomes based agreement for this medicine, but it was not a proposal from the IPT |

**Table 56** **Checklist for assessing gene therapies. Drug: talimogene laherparepvec (T-VEC) (Imlygic) for treating unresectable metastatic melanoma**

**Setting: Spain – National Level – IPT (Therapeutic Positioning Report)**

| Item | Yes | No | Notes |
| --- | --- | --- | --- |
| Surrogate endpoint used | X |  | **Validation given?** No  Durable response rate (DRR) is the primary endpoint of the pivotal clinical trial (OPTiM); overall survival is the key secondary endpoint. |
| Rare disease | N/A | | **Prevalence**: N/A  The IPT provided data about incidence (5.3 – 5.8 per 100,000) |
| Serious condition | X |  |  |
| Single-arm trial |  | X | **Matched historical cohort used?** No  Head to head versus GM-CSF (Granulocyte Macrophage Colony-Stimulating Factor) |
| Pediatric population |  | X | **Age range**: adults (age ≥ 18 years) |
| Reporting of adverse consequences and risks | X |  | The authority expressed concerns on lack of data supporting the long-term safety of the treatment |
| Size of clinical trial | **Number of patients**: 436 | | 1. + 141 (control arm) Study 005/05 |
| Length of clinical trial | **Duration in months**: | |  |
| Extrapolation to long-term outcomes | **Duration in months**: | |  |
|  | **Yes** | **No** | **Quantification** |
| Severe disease | X |  |  |
| Value to caregivers | N/A | |  |
| Insurance value | N/A | |  |
| Scientific spillovers | N/A | |  |
| Lack of alternatives |  | X | According to the IPT, there are alternatives |
| Substantial improvement in life expectancy | X |  |  |
|  | **Yes** | **No** | **Notes** |
| Discounting |  | X |  |
| Different discount rates explored |  | X |  |
| Uncertainty |  | X |  |
| Alternative payment models explored |  | X | THIS MEDICINE IS NOT REIMBURSED IN SPAIN |
|  |  |  |  |

**Table 57 Checklist for assessing gene therapies. Drug: voretigene neparvovec (Luxturna) for treating inherited retinal dystrophies caused by rpe65 gene mutations.**

**Setting: Spain – National Level – IPT (Therapeutic Positioning Report)**

| Item | Yes | No | Notes |
| --- | --- | --- | --- |
| Surrogate endpoint used | X |  | **Validation given?** N/A  Visual acuity in the primary endpoint |
| Rare disease | X |  | **Prevalence**: 3.7 per 100,000 people (hereditary retinal dystrophies) |
| Serious condition | X |  | Large unmet need stated by manufacturer, patient organizations, and expert opinion. The authority did not dispute the claim. |
| Single-arm trial |  | X | **Matched historical cohort used?** No |
| Pediatric population | N/A | | **Age range**: N/A  The drug is licensed for both pediatric and adult population. However, mean age in the pivotal clinical trial was 15.1 years, reflecting early onset of the disease. |
| Reporting of adverse consequences and risks | X |  |  |
| Size of clinical trial | **Number of patients**: 29 | | Studio 301. |
| Length of clinical trial | **Duration in months**: | |  |
| Extrapolation to long-term outcomes | **Duration in months**: N/A | | Lack of long term data |
|  | **Yes** | **No** | **Quantification** |
| Severe disease | X |  | . |
| Value to caregivers |  | X |  |
| Insurance value | N/A | | N/A |
| Scientific spillovers |  |  | N/A |
| Lack of alternatives | X |  |  |
| Substantial improvement in life expectancy |  | X | Survival is not a relevant endpoint in this disease |
|  | **Yes** | **No** | **Notes** |
| Discounting |  | X |  |
| Different discount rates explored |  | X |  |
| Uncertainty | X |  | Lack of Long term data and safety |
| Alternative payment models explored |  | X | This medicine is under a outcomes based agreement |
